# Controlling Exciton Propagation in Organic Crystals through Strong Coupling to Plasmonic Nanoparticle Arrays

**DOI:** 10.1021/acsphotonics.2c00007

**Published:** 2022-06-09

**Authors:** Anton Matthijs Berghuis, Ruth H. Tichauer, Lianne M. A. de Jong, Ilia Sokolovskii, Ping Bai, Mohammad Ramezani, Shunsuke Murai, Gerrit Groenhof, Jaime Gómez Rivas

**Affiliations:** † Department of Applied Physics and Eindhoven Hendrik Casimir Institute, Eindhoven University of Technology, P.O. Box 513, 5600 MB Eindhoven, The Netherlands; ‡ Nanoscience Center and Department of Chemistry, University of Jyväskylä, P.O. Box 35, 40014 Jyväskylä, Finland; § Department of Material Chemistry, Graduate School of Engineering, Kyoto University, Katsura, Nishikyo, 6158510, Kyoto, Japan; ∥ Institute for Complex Molecular Systems ICMS, Eindhoven University of Technology, P.O. Box 513, 5612 AJ, Eindhoven, The Netherlands

**Keywords:** strong light−matter coupling, polariton transport, molecular dynamics simulations, tetracene, plasmonics, nanoparticle array

## Abstract

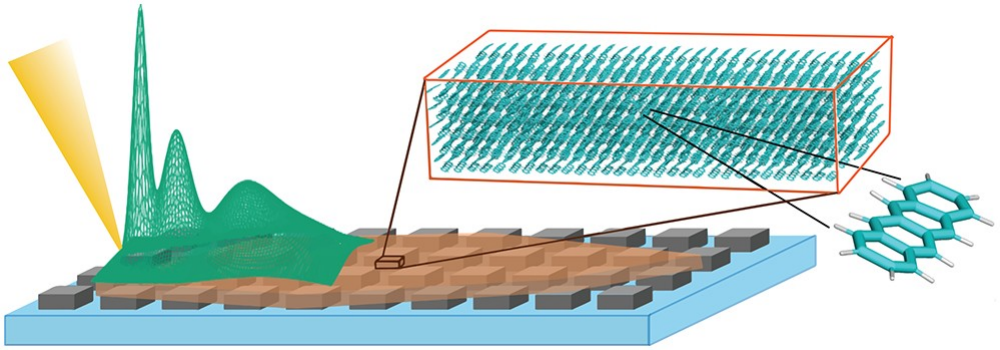

Exciton transport in most organic materials is based on an incoherent hopping process between neighboring molecules. This process is very slow, setting a limit to the performance of organic optoelectronic devices. In this Article, we overcome the incoherent exciton transport by strongly coupling localized singlet excitations in a tetracene crystal to confined light modes in an array of plasmonic nanoparticles. We image the transport of the resulting exciton–polaritons in Fourier space at various distances from the excitation to directly probe their propagation length as a function of the exciton to photon fraction. Exciton–polaritons with an exciton fraction of 50% show a propagation length of 4.4 μm, which is an increase by 2 orders of magnitude compared to the singlet exciton diffusion length. This remarkable increase has been qualitatively confirmed with both finite-difference time-domain simulations and atomistic multiscale molecular dynamics simulations. Furthermore, we observe that the propagation length is modified when the dipole moment of the exciton transition is either parallel or perpendicular to the cavity field, which opens a new avenue for controlling the anisotropy of the exciton flow in organic crystals. The enhanced exciton–polariton transport reported here may contribute to the development of organic devices with lower recombination losses and improved performance.

## Introduction

Energy transport is a crucial process in organic optoelectronic devices, such as organic photovoltaics (OPV) or organic light-emitting diodes (OLEDs). Because excitons in organic semiconductors (Frenkel excitons) have large binding energies and are predominantly localized onto single molecules, exciton transport proceeds via incoherent hopping. This hopping process is hampered by thermal and structural disorder, which limits exciton diffusion lengths to values below 10 nm for most materials.^1^

Even in organic crystals with suppressed structural disorder, such as tetracene (Tc), the singlet exciton diffusion length is on the order of 50 nm only,^2^ though it can be increased by an order of magnitude through dark triplet states.^3^ The short diffusion lengths represent a bottleneck for the development of optoelectronic devices as they require complex morphologies of active layers in nanometer sized domains, for example, bulk heterojunctions in OPV, which not only complicate the fabrication, but also reduce device stability.^4,5^

To overcome limitations related to short exciton diffusion lengths in organic materials and increase the distance over which energy can be transported, it has been proposed to strongly couple Frenkel excitons to the confined light modes of an optical cavity.^6,7^ In this light–matter interaction regime, excitons and optical modes hybridize into new light–matter states, called polaritons.^8−10^ Being coherent superpositions of material excitations and confined photons, polaritons not only have a very small effective mass but also possess group velocity, which can be exploited to transfer polaritonic wavepackets over long distances. Indeed, in an early study from 2000, inorganic exciton polaritons displayed ballistic propagation within an InGaAs quantum well placed inside an optical microcavity.^11^ Later, long-range exciton polariton propagation was also experimentally demonstrated for organic media where excitons were coupled to Bloch surface waves^12,13^ and for plasmonic nanoparticle arrays coupled to excitons in carbon nanotubes.^14^ A much smaller enhancement of the transport length was achieved for Frenkel excitons strongly coupled to confined light modes of an optical microcavity^15^ and for polaritons in cavity-free systems,^16^ where the polaritons transport mechanism appeared to be (partially) diffusive. The reason for the different behavior of polaritons is not clear as a description of the underlying processes on the molecular/excitonic level is missing, leaving the polariton-enhanced transport mechanism open for interpretation.

In this Article, we demonstrate improved exciton–polariton transport via strong coupling of excitons in tetracene (Tc) to surface lattice resonances (SLRs) in open cavities formed by nanoparticle arrays. We also employ multiscale Molecular Dynamics (MD) simulations to unravel the properties of the propagating polaritons.

SLRs are optical modes in nanoparticle arrays that arise from the interaction of the localized surface plasmon resonances and the in-plane diffractive orders. SLRs have controllable dispersion, enabling the engineering of long-range transport of excitations.^17−24^ Moreover, owing to the open architecture of plasmonic lattices, SLRs are easy to integrate with organic semiconductors.^25,26^ We selected Tc crystals as the excitonic material because of its intrinsic long exciton diffusion length and promising properties in upconversion photovoltaics.^27−29^

We achieve strong coupling by aligning the transition dipole moment of Tc excitons, oriented along one of the axes of the crystal, to the SLR field and investigate the propagation of the coupled exciton–polaritons. We observe that polariton transport depends strongly on the relative orientation between the transition dipole moment of Tc and the SLR field, demonstrating selective transport defined by the nanoparticle array. Experimental observations are reproduced by atomistic multiscale MD simulations, providing further support that also the excitonic component of polaritons propagates over long distances. We find that the propagation length of Tc singlet excitons coupled to SLR modes is 2 orders of magnitude larger than the diffusion length of singlet excitons for exciton–polaritons with an exciton fraction of 50%. This remarkable enhancement and the possibility to control it by the relative orientation between the crystal and the nanoparticle array opens new opportunities for controlling exciton flows in organic devices.

## Results and Discussion

### Cavity Design

**Figure 1:**
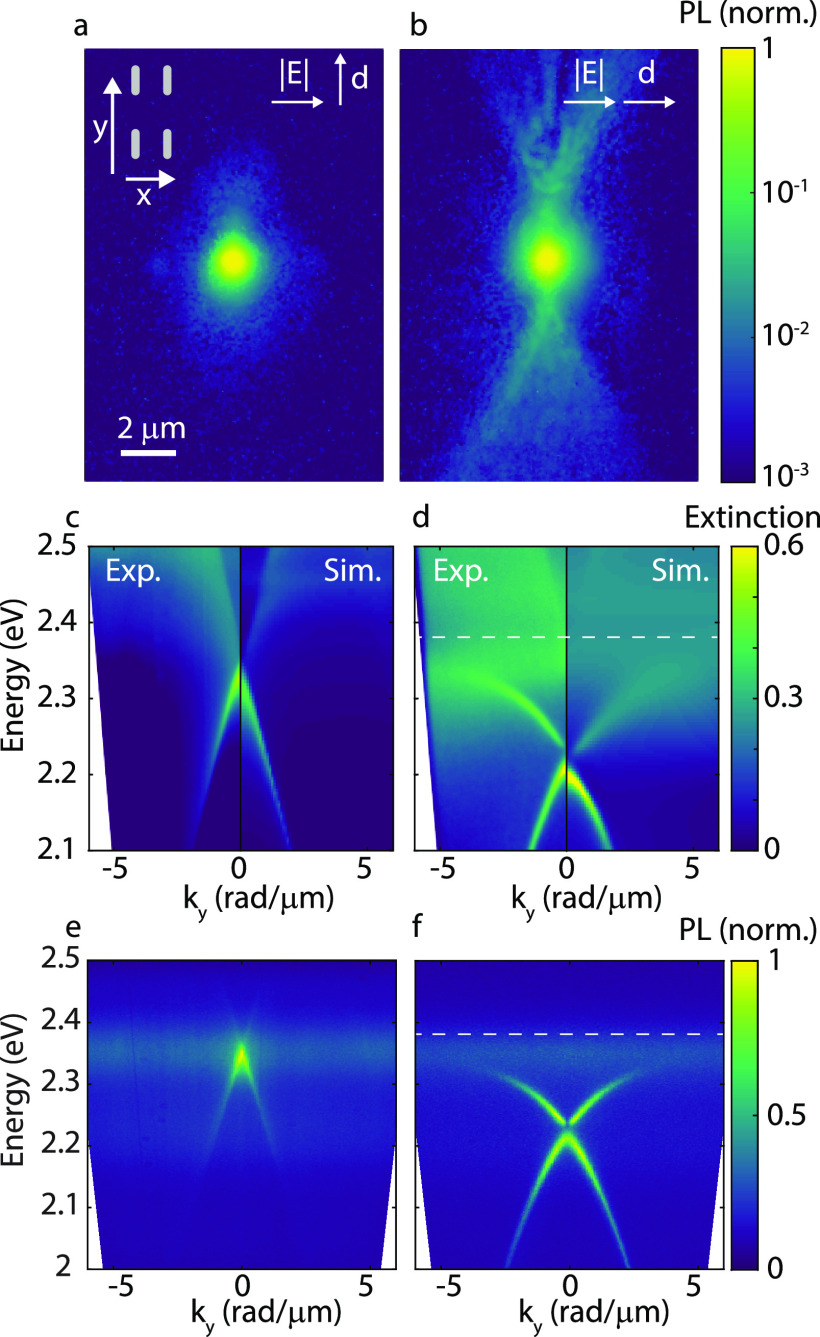
Fluorescence images following a focused laser excitation of a Tc crystal placed on top of the plasmonic cavity (a and b), with the S_0_ → S_1_ transition dipole moment in Tc oriented perpendicular (weak coupling regime) (a), and parallel (strong coupling regime) (b) to the SLR field. Dispersion of the extinction along the propagation direction (*k*_*y*_) of the weakly (c) and strongly (d) coupled system. The left panel of each figure displays the experimental data, while the right panels are results from the FDTD simulations. Emission for the weakly (e) and strongly (f) coupled system. Strong coupling is evidenced in (d) and (f) by the bending of the exciton–polariton band away from the exciton energy at 2.38 eV.

We have designed a cavity of silver nanoparticles with a lattice constant of 240 × 360 nm^2^ and a particle size of 42 × 100 nm^2^ (see Supporting Information (SI) Figure S1). The individual nanoparticles support localized surface plasmon resonances (LSPRs) with a fundamental energy of ≃2.7 eV for a polarization along the short axis (see SI, Figure S1). These localized resonances couple to the in-plane diffraction orders, resulting in transverse electric (TE) and transverse magnetic (TM) surface lattice resonances. In this manuscript we investigate SLRs corresponding to the first diffraction order along the *y*-direction (i.e., the (0,–1) and (0,+1) orders), corresponding to a period of 360 nm (see Figure 1; see SI, S1 for a description of the terminology). We focus on the TE mode, as this mode has a larger group velocity than the TM mode, especially at low k-vectors.

### Propagation and Dispersion Characterization

A saturated solution of tetracene (99.99% Sigma-Aldrich) in toluene was dropcasted on the nanoparticle array, resulting in the growth of thin crystals (50–200 nm) with relatively large lateral dimensions (up to 500 μm), as described in refs (30 and 31). Then, a small region of the Tc crystal, placed on top of the cavity, was excited by a focused laser beam (λ = 450 nm). We measured the emitted light from the coupled system with a confocal microscope (Nikon Ti-Eclipse with 100x 0.9 NA objective lens). The fwhm of the detected laser spot is approximately 900 nm, which after deconvolution with the point spread function of our system ∼400 nm, comes down to a spot size of ∼800 nm. When the transition dipole moment in Tc is oriented orthogonal to the cavity field, we observe negligible propagation, as shown in Figure 1a. However, propagation over several micrometers is observed when the crystal is rotated such that the dipole moment is aligned to the field associated with the SLR defining the cavity (Figure 1b). The slight asymmetry of the observed propagation is due to a few degrees misalignment between the cavity field and the dipoles of Tc excitons.

To investigate the propagation of exciton-polaritons in more detail, we have measured their dispersion by retrieving the angle-resolved extinction and emission spectra using Fourier microscopy. When the SLR field and the dipole moment are orthogonal, the dispersion of the TE mode is very similar to that of the nanoparticle array defining the bare cavity (see SI, Figure S1), that is, for energies much lower than the LSPR (∼2.7 eV), the dispersion appears as straight lines, as described by the grating equation (left panel of Figure 1c). This indicates that there is no strong coupling between the SLR cavity mode and the Tc exciton resonance. We compare the experimental results with a finite-difference time-domain (FDTD) simulation of the nanoparticle array covered with a 140 nm thick Tc crystal for which we use the dielectric tensor, as measured by Tavazzi et al.^32^ (SI, Figure S3). The simulated dispersion, plotted on the right panel of Figure 1c for positive in-plane momentum *k*_*y*_, shows an excellent agreement with the experimental data (note that due to the 180° rotational symmetry, the system is invariant under reflection over *k*_*y*_ = 0).

When the dipole moment of Tc excitons and the cavity field are oriented parallel, there is a clear bending of the mode away from the exciton transition energy at 2.38 eV, indicating the formation of exciton–polariton states and strong light–matter coupling (left panel of Figure 1d). The experimental results are verified by FDTD simulations, as plotted in the right panel of Figure 1d. An interesting feature in these plots is the gap in the dispersion at *k*_*y*_ = 0, where the (0,+1) and (0,–1) TE modes cross. This splitting corresponds to symmetric (bright) and antisymmetric (dark) field distributions of the modes.^26,33^ At this crossing point, the group velocity of the modes is much lower, which leads to a reduced propagation length, as we will show later. The dispersion measurements and simulations show a very clear lower polariton band (LPB), while the upper polariton band (UPB) is hardly visible, which could be explained by the additional coupling to higher vibronic modes. The UPB is visible with TM polarization only at higher k-vectors (SI, Figure S2) and shows a splitting of 220 meV, which confirms that this system is in the strong coupling regime.^31^

The effect of strong coupling is also clear in the modified dispersion of the emission spectra measured upon excitation by a 450 nm laser. The dispersion of the emission overlaps with the extinction for both the weakly (Figure 1e) and the strongly coupled cases (Figure 1f). While the emission is barely enhanced for the weakly coupled system, there is a very strong enhancement of the emission from the LPB for the strongly coupled system. This observation is in agreement with earlier studies^34^ and can be understood by the efficient internal conversion from excited states to the LPB.^35^

**Figure 2:**
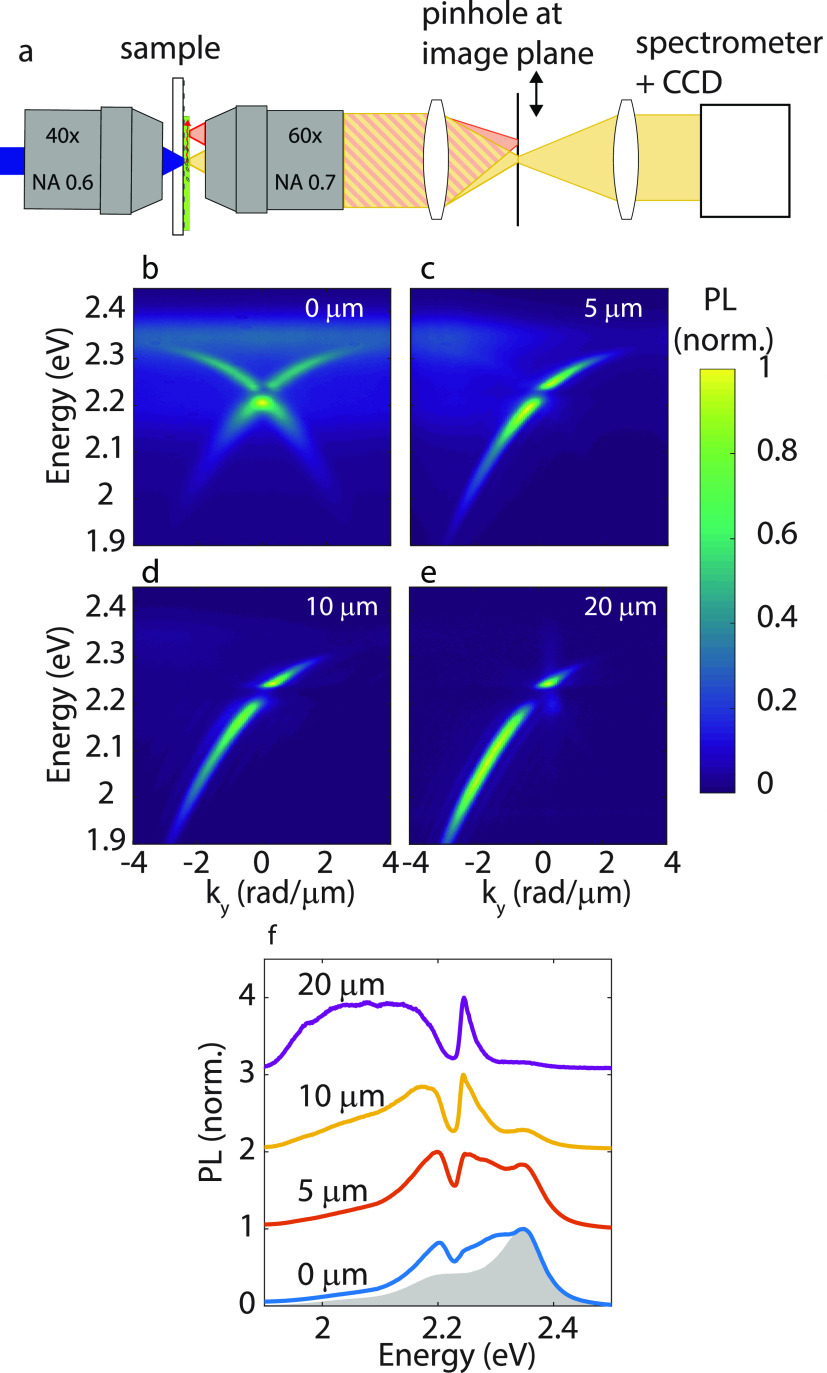
Schematic representation of the experiment (a). The strongly coupled Tc crystal is excited at position *y* = 0. The fluorescence is detected by selecting the emission from a small area (*d* = 6.7 μm) at a certain distance from the excitation spot by changing the position of a pinhole in an intermediate imaging plane. The back focal plane is mapped on the slit of a spectrometer that measures light with *k*_*x*_ = 0 via a grating on a CCD. Emission measured at different distances (0, 5, 10, and 20 μm) away from the excitation spot (b–e). Emission integrated over *k*_*y*_ for the four previous distances from the excitation spot (f). The gray shaded area corresponds to emission from uncoupled molecules.

In order to study the properties of the propagating LPB, we have imaged the Fourier plane of the emission at different distances from the excitation using a Fourier microscope with an intermediate imaging plane (40× excitation objective NA 0.6, 60× collection objective NA 0.7). For these measurements, we place a pinhole in the intermediate imaging plane corresponding to a 6.7 μm diameter spot on the sample (see setup illustration in Figure 2a). By choosing the position of the pinhole in the imaging plane, we can select the emission originating from a well-defined distance from the excitation. In Figure 2b–e, we image emission at distances of 0 to 20 μm away from the excitation spot where we normalize each image to its maximum intensity. The LPB modes are slightly less “sharp” than in Figure 1f, as the pinhole in the intermediate imaging plane leads to a lower resolution in the Fourier plane. At *y* = 0, the Fourier plane shows emission from both the (0,+1) and the (0,–1) LPB and emission from uncoupled excitons (Figure 2b). Moving the pinhole in the imaging plane away from the excitation spot in the +*y*-direction, the (0,–1) exciton-polariton mode, which propagates in the −*y*-direction, is not visible anymore and also the emission from uncoupled excitons is reduced (Figure 2c). Moving further away from the excitation spot, emission from uncoupled excitons disappears completely (Figure 2d,e). In addition to the vanishing emission of uncoupled excitons, the intensity distribution of the LPB emission changes as a function of the distance from the excitation spot, indicating an energy-dependent propagation length. At *y* = 5 μm from the excitation, the LPB is most intense at high energies (from 2.1–2.3 eV). Further away, at 10 and 20 μm, the highest intensity shifts to lower energies. In particular, the exciton–polariton at energies close to the Tc exciton energy disappears completely since it has a high exciton fraction and the lowest group velocity. Emission at *k* = 0 and 2.19 eV disappears as well in Figure 2d,e since the group velocity of the LPB is zero due to the interaction between the forward and backward propagating TE modes, forming a standing exciton–polariton mode. To stress further these effects, the spectra of the emission integrated over the wave vector are plotted in Figure 2f and compared to the fluorescence of the uncoupled Tc (shown by the gray shaded area in the same figure). The red shift of polariton emission as the distance from the excitation increases is very clear in this figure. Despite this red shift, it should be noted that for all wavelengths the emission intensity decreases as a function of distance from the excitation spot. We will quantitatively discuss the propagation length in Figure 4, but first we discuss the underlying molecular processes of polariton transport by means of MD simulations.

### Molecular Dynamics Simulations

**Figure 3:**
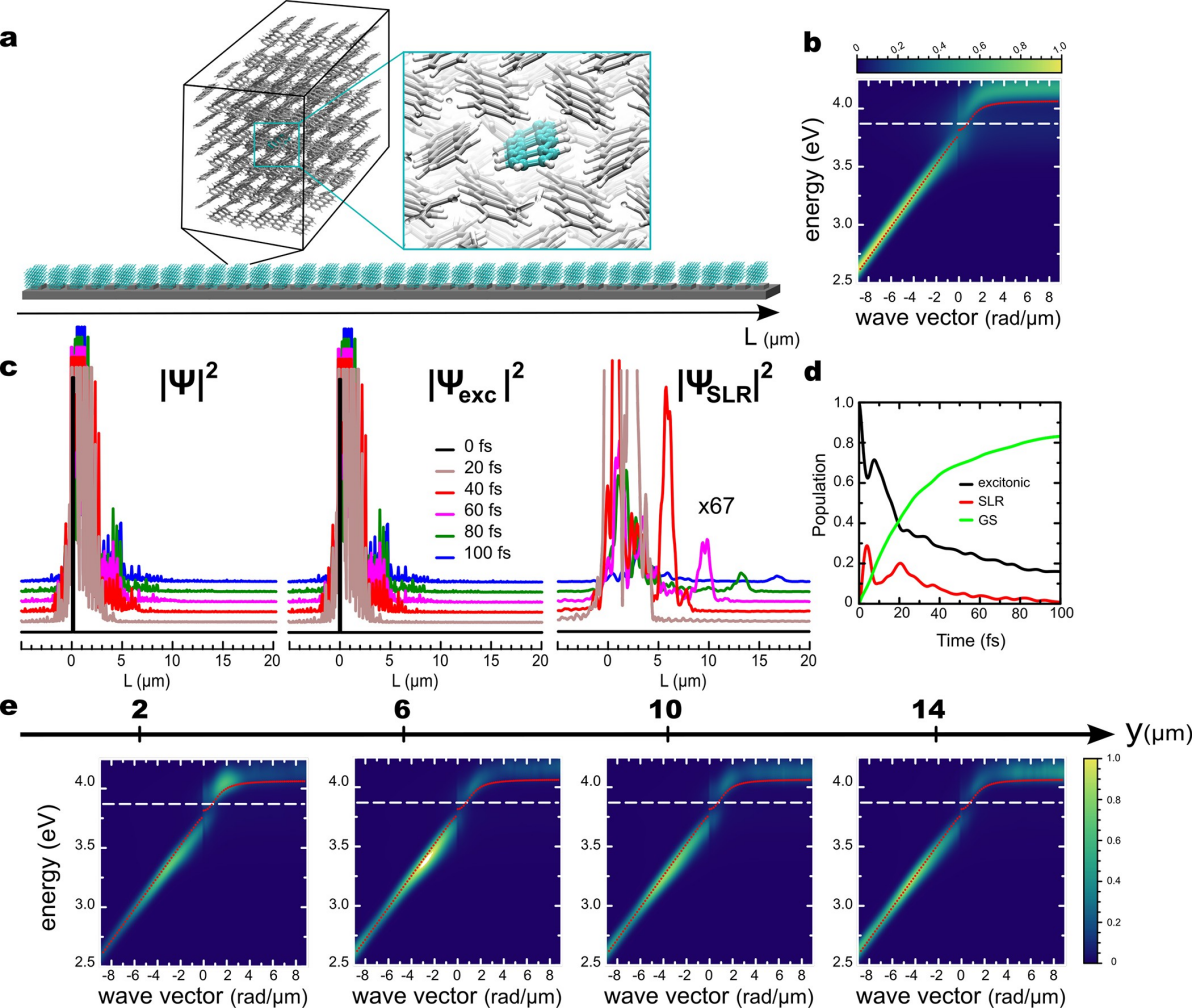
Schematic illustration of the simulation setup with the insets showing one of the 512 unit cells modeled in MD simulations (a). The Tc monomer described at the QM level is shown in ball-and-stick representation, while monomers in the MM subsystem are represented by sticks. Angle-resolved absorption spectrum when the crystal is strongly coupled to the modes of the (0,+1) SLR (b). Wave-packet motion along the +*y*-direction (c). For clarity, a vertical offset is added to wave packets at different time points. Time evolution of the excitonic (black) and photonic (red) fractions of polaritons in the Tc-SLRs strongly coupled system, as well as population of the ground state occupation (green), in which no photon is present (d). Angle-resolved photoluminescence spectra observed through pinholes located at various distances along the *y*-axis (e). Intensities in these plots are scaled by the same factor for each pinhole.

To obtain atomistic insights into polariton propagation, we performed multiscale Quantum Mechanics/Molecular Mechanics (QM/MM) MD simulations of a Tc crystal strongly coupled to the (0,+1) SLR mode in a periodic one-dimensional lattice of length 36 μm (Figure 3a). Because we are interested in the propagation along the +*y*-direction, the (0,+1) SLR was modeled only.

In line with observations from the imaging experiments, also the MD simulations suggest a rapid propagation of exciton–polaritons over several micrometers after nonresonant excitation into a single Tc monomer of the crystal. Figure 3b shows the absorption of the Tc crystal interacting with the (0,+1) SLR as a function of the wave vector. The avoided crossing between the upper and lower absorption branches suggests that the system is in the strong coupling regime, with a Rabi splitting of ∼250 meV. Because the nuclear degrees of freedom are described classically in our simulations, we can only couple the vertical S_0_ → S_1_ transition without vibronic progression. Therefore, the UPB is clearly visible in our simulations, in contrast to the experiment.

As shown in Figure 3c and in animations provided in the SI, the wave packet Ψ(*t*) spreads out during the simulations, covering an increasing area of the Tc crystal with time. This panel also resolves how much excitons in each Tc unit cell contribute to the amplitude of |Ψ(*t*)|^2^. These contributions are manifested by the spikes that indicate where excitons are transiently located during the wave packet propagation. The finite lifetime of SLR modes which establishes a competing channel to polariton propagation through radiative decay into the overall ground state (green curve in Figure 3d), restricts the maximum propagation length in our simulations to below 20 μm in 100 fs (Figure 3c and Figures S8(c) and S9(c) in the SI). While this distance is independent of the number of Tc unit cells in our simulations, we observe that the wave packet survives longer with increasing number of Tc crystal unit cells (see animations provided as SI). The latter dependency can be explained by the density of dark states, which increases with the number of strongly coupled Tc unit cells. Because these dark states lack a SLR contribution, the dark state manifold acts as a reservoir that extends the lifetime of the strongly coupled system.^36,37^ While transient population of dark states increases the lifetime in our system, we note that if the decay of the confined light modes were much slower than that of the exciton, the lifetime of dark states would become the limiting factor in the propagation process, as observed for Bloch Surface Wave Polaritons.^12,13^

Closer inspection of the MD trajectories reveals that relaxation of the photoexcited Tc molecule induces a transfer of population into polaritonic states, as manifested by the increase of the SLR mode contribution in Figure 3d. These polaritonic states then propagate along the + *y* direction with their group velocities. Even if propagation is ballistic, it appears diffusive because (i) the group velocities of polaritonic states span a wide range (Figure S10 in the SI), with bright states propagating at their respective group velocity and stationary dark states not propagating at all, and (ii) reversible population transfers between these states. Because the group velocities are higher for polaritonic states at lower energies, but level off at higher energies where the polaritonic states are dominated by excitonic contributions, the SLR-dominated lower energy wave-packets propagate faster, and reach a longer propagation distance, despite their shorter lifetime. In Figure 3, this is manifested by *(i)* a narrow peak in the plot of |Ψ_SLR_(*t*)|^2^ that travels ahead of the rest of the wave packet (Figure 3c) and by *(ii)* a red-shift in the maximum intensity of the photoemission at further distances (Figure 3e). The latter observation is in agreement with the dispersion measurements and suggest that the MD simulations capture the polariton dynamics, at least qualitatively.

### Propagation Length

**Figure 4:**
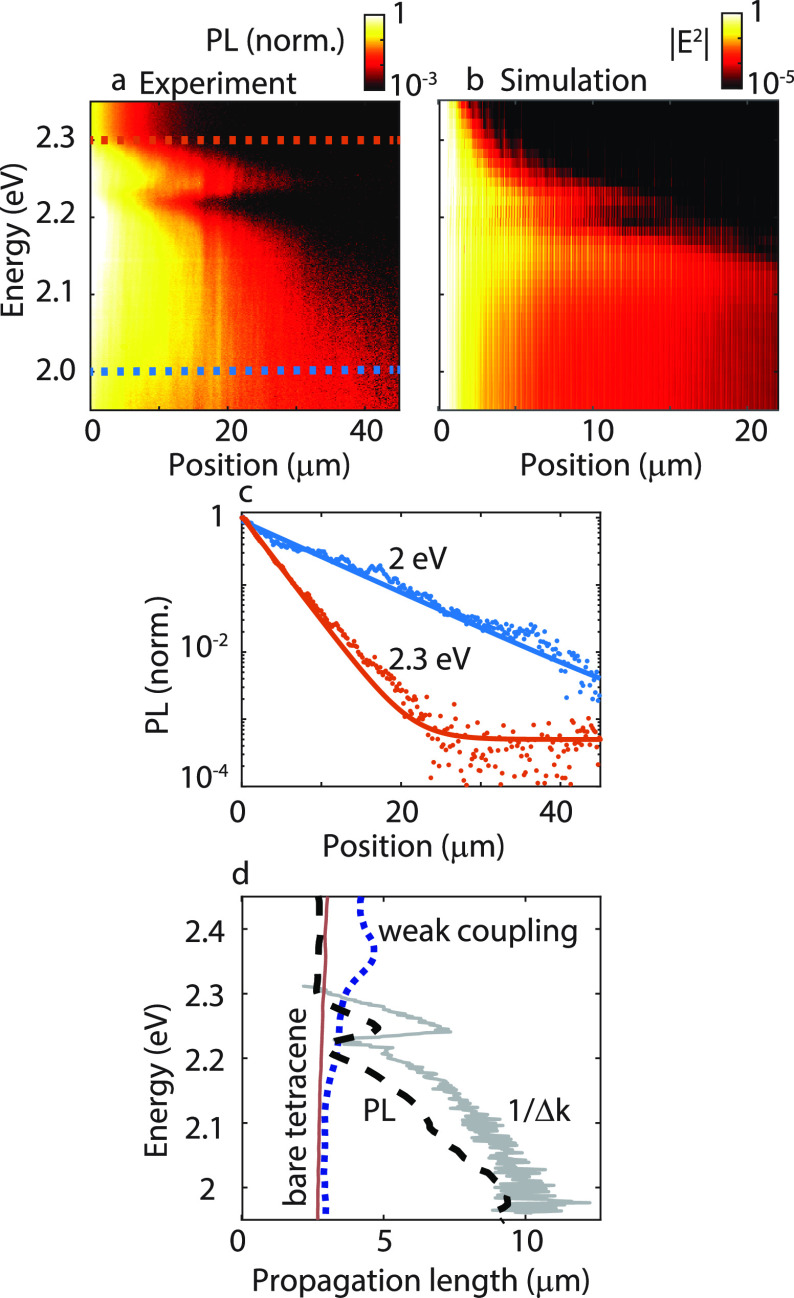
Real space map of the propagation of exciton-polaritons as a function of energy, normalized by the intensity at a distance of 2 μm away from the laser excitation (a). FDTD simulation of the propagated squared electric field intensity |*E*|^2^ in an array of 61 × 25 particles with a dipole at *y* = 0 (b). Cross section of the emission intensity as a function of distance from the excitation spot (c) for an energy of 2 eV (blue circles) and 2.3 eV (orange circles), corresponding to the horizontal lines in (a). The solid lines are exponential fits to the data. The propagation length obtained from fitting the decay at energies between 1.95 and 2.45 eV is plotted in (d) with the black dashed curve. This curve matches excellently with the propagation length obtained from the dispersion of Figure 1f as 1/Δ*k*_*y*_, given by the gray curve. The red curve corresponds to the measured propagation length in the Tc crystal in absence of the particle array.

To quantify experimentally the propagation length of exciton–polaritons as a function of energy, we map the emission intensity as a function of the distance from the laser excitation (Figure 4a). To reduce the contribution of scattered uncoupled emission of Tc, we follow the method proposed by Zakharko and co-workers:^14^ We excite the crystal 2 μm away from the edge of the array, and normalize the measurements to the emission intensity at the edge. We also verify the observed exciton-polariton propagation using FDTD simulations. For these simulations, we consider a system of 25 × 61 silver particles covered with Tc and place a radiating point dipole that simulates the focused laser excitation, at the edge of this finite array. The dipole is oriented with a moment along the dominant SLR electric field component in the center of four particles (see SI, Figure S5). This choice is motivated to increase the coupling of the emission to the SLR and to reduce the quenching of this emission by avoiding the near-field coupling to higher order multipoles in the nanoparticles. The square of the electric field amplitude |*E*|^2^ in the Tc crystal obtained by FDTD simulations, is plotted in Figure 4b after normalization at a position 1.5 μm away from the dipole to exclude the near-field contribution of the point dipole emission. The simulated exciton–polariton propagation agrees qualitatively with the experimental propagation data. This simulation shows an increasing value of the propagation length for low energies and a similar dip in this propagation length at approximately 2.2 eV, where the (0,+1) and (0,–1) polaritons cross. It should be noted, however, that the decay of |*E*^2^| in the FDTD simulation is roughly a factor of 2 faster compared to the experiment as can be appreciated from the different color scales used in Figure 4a,b and from the cross sections plotted in SI, Figure S5. This quantitative discrepancy between simulations and experiments could be due to a reduction of the quality factor of the resonances in the simulations due to the limited dimension of the array,^38^ and the fact that a single point dipole can not fully capture the effects of the laser excitation.

We fit the experimental data of the decaying fluorescence with an exponential function , where *L*_p_ is the propagation length, and *y* is the distance from the excitation source. The emission intensity decay and the fit to the data are given in Figure 4c for an energy of 2.3 eV, that is, close to the exciton resonance (orange circles and curve), and for 2 eV, that is, far away from the exciton resonance (blue circles and curve), clearly showing the difference in propagation length. In Figure 4d, we show with a black dashed curve the results of the fits to the decay of the fluorescence intensity as a function of the energy. We also obtain the propagation length from the far-field emission as the inverse of the imaginary component of the wave vector (1/Im(*k*_*y*_)) of the LPB, corresponding to 1/Δ*k*_*y*_^39,40^ for each energy (gray curve in Figure 4d). This line width is determined by fitting a Lorentzian function to the emission spectrum of Figure 1f. The propagation length for the strongly coupled case is clearly much larger than for the case when the cavity field and Tc dipoles are orthogonal, as appreciated when comparing the propagation lengths obtained from the real space images of the fluorescence and plotted with the blue dotted curve in Figure 4d. The origin of the propagation for the weakly coupled system is the coupling of emission into the SLR, followed by out-coupling at a different position.

The red curve in Figure 4d indicates the propagation length of the exciton in the Tc crystal outside of the array (including internal reflections in the substrate), where the propagation is either due to the diffusion of triplets that annihilate into singlets^3,41^ or to the emission and reabsorption of the fluorescence.

**Figure 5:**
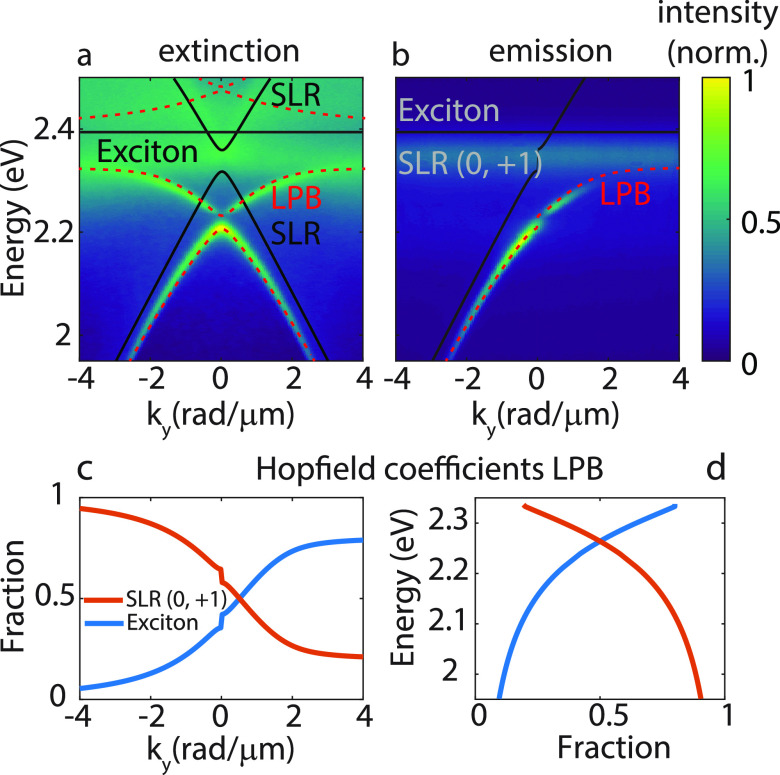
Dispersion measurements and fitted dispersion (red dashed curves) of the strongly coupled system with a coupled oscillator model (a). The black curves are the dispersion of the uncoupled SLR and exciton. Fluorescence map obtained by exciting the system at the edge of the array in order to only excite the (0,+1) TE SLR (b). The fluorescence follows the same dispersion as the extinction map. The SLR fraction (orange curve) and exciton fraction (blue curve) of the LPB as obtained by the coupled oscillator model is shown in (c) as a function of the in-plane momentum and in (d) as a function of LPB energy.

The exciton–polariton propagation length, as determined from the dispersion measurements and the real space emission spectra, are in excellent agreement and show a propagation length of 9 μm at 1.95 eV. Low-energy polaritons have a high photonic content, which explains such a long propagation distance. This length is only a few microns shorter than the propagation length of the bare SLR, which propagates up to 15 μm at low energies (see SI, Figure S1(e)). The excitonic/photonic fraction of exciton–polaritons are given by the mixing coefficients that can be estimated by fitting the dispersion measurements to the Hamiltonian of the coupled system,^42^
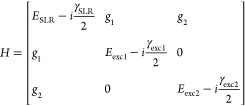
1where *E*_SLR_ is the angle-dependent energy dispersion of the SLR, which is obtained through the coupling of the LSPR and the Rayleigh Anomalies (RAs), as obtained with a similar coupled model and plotted in Figure 5a with the black curve. γ_SLR_ are the losses of the SLR, which depend on the in-plane momentum as they increase when the cavity dispersion approaches the LSPR energy (SI, S7). *E*_exc1_ and *E*_exc2_ are the two excitonic transitions of Tc, that is, the S_0_ → S_1_ transition and the vibronic progression, centered at 2.38 and 2.57 eV, respectively, with their losses estimated from the fwhm of the spectrum, yielding γ_exc1_ = γ_exc2_ = 140 meV. The exciton energy of 2.38 eV is indicated in Figure 5a with the horizontal black line. The coupling strengths between the SLR and the exciton transitions are denoted by *g*_1_ and *g*_2_, respectively, with *g*_1_ = 160 meV and *g*_2_ = 60 meV. The fact that *g*_1_ is larger than the losses indicates that the system is in the strong coupling regime.^43^ The diagonalization of this matrix gives the eigenvalues and eigenvectors of the coupled system. The eigenvalues are plotted with the red dashed curves in Figure 5a as a function of the in-plane wave vector.

For propagation in the +*y*-direction, we are only interested in the LPB since emission from the middle and upper polaritons cannot be detected due to fast internal conversion to dark states and LPB. We only look at the propagation along the +*y*-direction and therefore focus on the TE LPB mode. We can visualize this mode when the Tc crystal is excited at the edge of the particle array and look at the fluorescence in the Fourier plane. In Figure 5b, we can see that this mode overlaps with the fitted TE polariton mode (red-dashed curve). As a reference, the exciton transition at 2.38 eV and the uncoupled SLR are plotted with the black line and curve in the same figure.

The exciton fraction and SLR fraction of the different polariton modes are given by the mixing coefficients that are obtained by squaring the amplitude of the eigenvectors.^44^ The mixing coefficients for the (0,+1) TE LPB are plotted in Figure 5c as a function of the in-plane wave vector. The exciton fraction of the (0,+1) mode (blue curve in Figure 5c) increases as LPB approaches the exciton transition at larger wave vectors, while the SLR fraction decreases (orange curve). The small jump in both curves at *k_y_*= 0 corresponds to the gap in the LPB due to the coupling of the (0,+1) and (0,–1) SLRs that is visible in Figures 1d and 5a. The SLR and exciton fractions as a function of LPB energy are plotted in Figure 5d, showing the reduction of the SLR fraction and increase of the exciton fraction as the LPB approaches the exciton energy.

**Figure 6:**
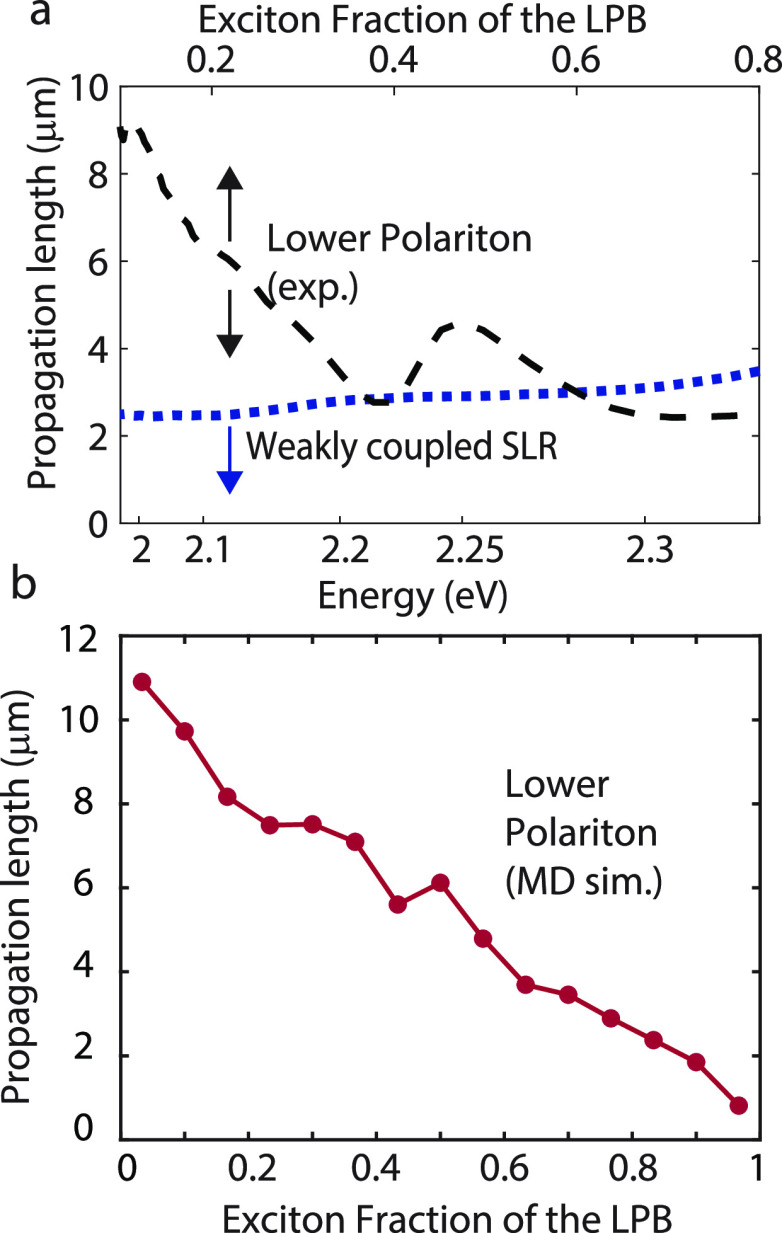
(a) Propagation length as a function of exciton fraction of the LPB (black dashed curve), obtained by combining the propagation length for each energy (Figure 4c) and the exciton fraction of the LPB as a function of energy (Figure 5d). A propagation length up to 9 μm is reached for low exciton fractions, but even at an exciton fraction of 0.5, the propagation length is 4.4 μm. The dip in propagation length at an exciton fraction of 0.4 corresponds to the gap in the dispersion of the LPB. The blue dashed curve shows the measured propagation length of excitons in a weakly coupled Tc crystal, that is, a crystal with the dipoles perpendicular to the cavity field. (b) Propagation length as a function of exciton fraction of the LPB obtained from the MD simulations.

We are mostly interested in the exciton fraction that gets transported through the LPB mode. Combining the data of Figures 5d and 4d, we obtain the propagation length as a function of exciton fraction, which is plotted with the black dashed curve in Figure 6a. In general, this curve shows a decreasing trend for higher exciton fractions, which is to be expected due to the localized character of excitons. The reduced polariton propagation at the crossing point of the forward and backward propagation SLRs at *k_y_* = 0, is visible as a dip in the propagation length at 2.21 eV. From the figure we can see that at an exciton fraction of 50%, the propagation length is 4.4 μm. In the same figure, we also plot the propagation length as measured for the weakly coupled system (blue dotted curve). The much shorter propagation length when the dipoles are orthogonal illustrates again the effect of the relative dipole orientation on the transport properties of the system. We speculate that the measured propagation length in the weakly coupled crystal is due to singlet fission resulting in long-lived triplet states followed by triplet–triplet annihilation at a distance from the excitation spot or scattering of the pump laser.

A similar trend is observed when plotting the propagation length as a function of exciton fraction from MD simulations (Figure 6b). However, in contrast to experiment, the minimum propagation length goes to zero with increasing exciton fraction. This difference arises because the initial excitation in the simulation is localized on a single QM subsystem and the purely excitonic states do not display a diffusive behavior because neither singlet–triplet intersystem crossing, nor excitonic couplings governing incoherent hopping of excitons between adjacent Tc monomers are included in the MD model. While including the excitonic couplings in our simulation model is straightforward,^45^ a direct comparison to the experimental incoherent propagation length would not be meaningful as the time scales that we can reach in our simulations are on the order of hundreds of femtoseconds and hence much shorter than the time scales associated with incoherent hopping.

We note that the polariton propagation length in our simulations does not depend on the size of the crystal (compare wavefronts in panel (c) of Figures 3 and S8 and S9 of the SI). Therefore, the agreement between experiment and simulations support the interpretation that exciton transport is significantly enhanced under strong coupling between the singlet excitons and the SLR modes.

## Conclusions

The anisotropic properties of Tc make this organic semiconductor an ideal platform for studying polariton propagation. By choosing the orientation of the strongest excitonic transition dipole moment with respect to the field in an open plasmonic cavity formed by an array of Ag nanoparticles, we were able to switch from the weak to the strong coupling regime. In the strong coupling regime, we measured an exciton polariton transport length with a decay constant of 4.4 μm at 50% exciton fraction, that showed a strong dependence on the photonic fraction of the exciton–polaritons. Because the propagation enhancement is highest for the lower energy polaritons, exploiting such enhanced propagation requires an acceptor with an excitation energy matching the energies of these LPB states.

A one-to-one comparison of the ballistic transport length in strongly coupled Tc and the exciton diffusion length of uncoupled Tc crystals is not straightforward, as the ballistic transport is directional, while diffusion is a random walk process. We can however conclude that the exciton polariton propagation length is 2 orders of magnitude larger than the diffusion length of singlet excitons in Tc (*L*_D_ ≈ 50 nm),^2^ and a factor 10 larger than triplet mediated exciton diffusion *L*_D_ ≈ 560 nm.^3^ MD simulations qualitatively confirm the enhanced exciton-polariton transport and reveal that through relaxation of the photoexcited Tc molecule, exciton polaritons are populated and propagate with their respective group velocities until they decay at the rate determined by the finite lifetime of the open cavity. The wave-packets cover distances of several μm in 100 fs, which is orders of magnitude faster than bare exciton diffusion that proceeds on a ns to μs time scale.

## Methods

### Molecular Dynamics Simulations

Three sets of simulations were performed, in which the macroscopic crystal is modeled as 256, 512, and 1024 unit cells (see Figure 3a). Each unit cell of these crystals contains 250 Tc monomers, one of which is described at the QM level, while the other 249 Tc monomers are modeled with the Gromos96-54a7 molecular mechanics force field.^46^ The electronic ground state (S_0_) of the QM subsystem was modeled at the restricted Hartree-Fock level (RHF), while the configuration interaction method truncated to single excitations (CIS) was used to describe the first singlet excited state (S_1_). In both RHF and CIS calculations, the single-configuration electronic wave functions were expanded in the 3-21G basis set.^47^ The large system sizes in our simulations (up to 30720 QM and 7649280 MM atoms) necessitated this rather low level of theory, which resulted in an overestimation of the first singlet excited state by ∼1.4 eV.

Because we are interested in the dynamics of polariton transfer in the positive *y*-direction, we only considered the (0,+1) SLR and modeled this SLR with 101 modes equidistant in the *k*_*y*_-space (−8.73 rad μm^–1^ ≤ *k* ≤ 8.73 rad μm^–1^) in a periodic one-dimensional lattice with a length of 36 μm. A nonlinear fit to the experimentally determined dispersion of the SLRs was done to obtain an analytical expression for the SLRs dispersion *E*_SLR_(*k*_*y*_) used during the simulations (see exact procedure in the SI). To account for the systematic blue-shift of the Tc excitation energy due to the low level of QM theory employed in our simulations, a 1.44 eV energy offset was added to the SLR dispersion. The Tc crystal unit cells were placed directly on top of the plasmonic silver nanoparticles that were modeled implicitly as an inhomogeneous electric field with a spatial distribution that reflects the local field strength (see Figure S6 and details in section S8.1 of the SI). The finite SLR lifetime was included in the simulations as a *k*_*y*_-vector dependent first-order decay of the confined light modes (see Figure S7 and details in section S8.1 of the SI). We used the Ehrenfest, or mean-field, method to compute trajectories of the strongly coupled SLR–Tc system with a time step of 0.1 fs. The temperature was kept constant by coupling the simulation boxes to a thermal bath at 300 K, modeled by a stochastic thermostat.^48^ To model the nonresonant excitation with the pump laser, we started the simulations with the QM subsystem of the first unit cell (*j* = 1) in the first electronic excited singlet state (). A complete description of the simulations is included in the SI.

## Supporting Information Available

Animations of the propagating polaritonic wavepacket in the MD simulations (MP4)
Details on the bare nanoparticle array, strong coupling measurements of the TM mode, dielectric function of tetracene as used in FDTD simulations, propagation measurements on a weakly coupled sample, propagation from FDTD simulations, fits of the SLR dispersion, and detailed description of the MD simulations (PDF)
