# Supplementary material for: Controlling Exciton Propagation in Organic Crystals through Strong Coupling to Plasmonic Nanoparticle Arrays
Source: ACS Photonics. 2022 Jun 9;9(7):2263–72. doi: 10.1021/acsphotonics.2c00007 (PMC9306002; doi:10.1021/acsphotonics.2c00007)
Supplement: Supplementary file 2 — ph2c00007_si_002.pdf [file ph2c00007_si_002.pdf]

Supporting Information for "Controlling Exciton Propagation in Organic Crystals through Strong Coupling to Plasmonic Nanoparticle Arrays"

Anton Matthijs Berghuis, Ruth H. Tichauer, Lianne M.A. de Jong, Ilia Sokolovskii, Ping Bai, Mohammad Ramezani, Shunsuke Murai, Gerrit Groenhof, Jaime Gómez Rivas

Contents

S1 Fabrication and characterization of the bare nanoparticle array **S2**

S2 Strong coupling of tetracene to TM SLR **S3**

S3 Dielectric function of tetracene **S4**

S4 Propagation in weakly coupled tetracene **S4**

S5 Cross Section of the simulated and experimental propagation of the exciton polaritons **S5**

S6 Simulated Electric Fields **S6**

S7 Fitting of the dispersions of the SLRs of the bare array **S6**

S8 Molecular Dynamics Simulations **S7**

S8.1 Simulation Model S7
S8.2 Simulation Details S10
S8.3 Simulation Analysis S11
S8.4 Simulation results for different ensemble sizes S12
S8.5 Interplay between polariton group velocity and lifetime S14

S1 Fabrication and characterization of the bare nanoparticle array

The sample was fabricated using electron beam (EB) lithography and a lift-off process. A positive-type resist (ZEP520A) was spin-coated on an eagle glass substrate and the array patterns were written by EB lithography (F7000S-KYT01, Advantest, Japan). After development of the patterns by a developer solution (ZED-N50), a 3 nm-thick adhesion Ti layer and a 40 nm-thick Ag layer were deposited on the patterned resist by electron-beam deposition at room temperature. After that, the Ag nanoparticle pattern was formed by the lift-off of resist using a solvent (Microremover1165). Finally, the arrays were conformally coated with a 20 nm-thick aluminum oxide layer by atomic layer deposition (EALD-4, Eiko, Japan).

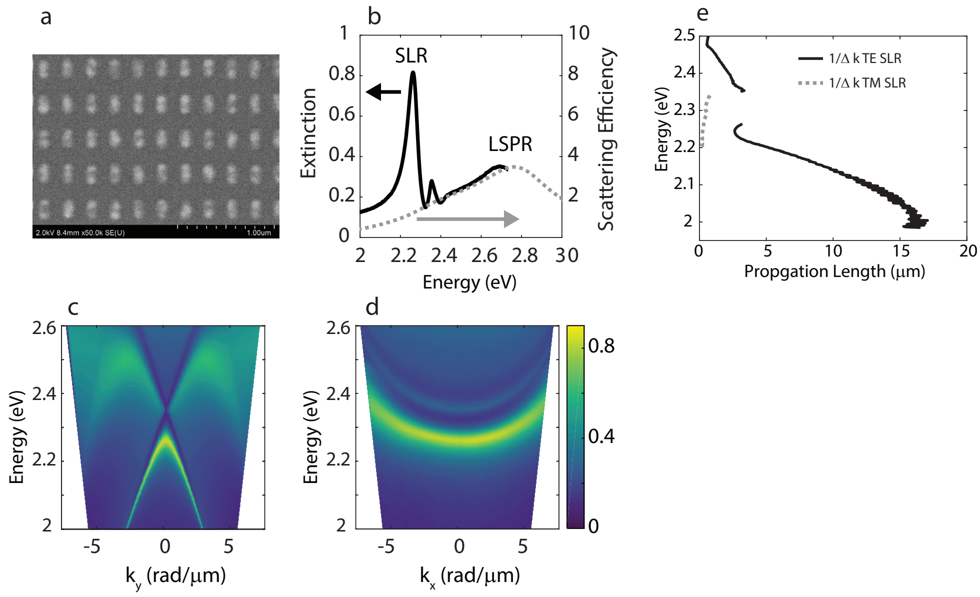

Figure S1: (a) Scanning electron microscope image of the silver nanoparticle array. The black curve in (b) shows the extinction of the particle array with a 200 nm PMMA layer on top and measured at normal incidence. The simulated scattering efficiency of a single particle is plotted with the dotted gray curve. The angular dispersion of the extinction of the array, when illuminated with light polarized along the short nanoparticle axis, is plotted in (c) and (d) for varying the in-plane momentum over k_y or k_x, respectively. (e) Propagation length of the bare TE and TM SLRs, obtained from fitting a Lorentzian function to the dispersions in S1 (c) and (d).

The array used in this manuscript has an array pitch of 240×360 nm^2 and size of ∼100×40×40 nm^3, as shown in the SEM Image Fig S1(a). The individual particles support a localized surface plasmon resonance (LSPR) with a maximum scattering efficiency in the measured range around 2.7 eV and when light is polarized along the short nanoparticle axis, as calculated by a total-field scattered-field FDTD simulation and plotted as the gray dotted line in Fig S1(b). When these particles are placed in an array and covered with a 200 nm PMMA layer, the coherent coupling of the LSPRs through the in-plane diffraction orders results in the formation of a surface lattice resonance (SLR), visible as a narrow peak in the extinction spectrum of the particle array at 2.26 eV and plotted with the solid black curve in Fig S1(b). The weaker peak at 2.35 eV is associated

to a guided mode in the PMMA layer.[1]

To describe the order of diffraction along the x and y directions we use the notation (p,q), as used in the equation for the Rayleigh anomalies,

$$E_{RA}(k_{||in})^2 = \left(\frac{\hbar c}{n}\right)^2 \left[{k_{||in}}^2 + \left(\frac{2\pi}{a_x}p\right)^2 + \left(\frac{2\pi}{a_y}q\right)^2 + 2k_x\frac{2\pi}{a_x}p + 2k_y\frac{2\pi}{a_y}q \right]. \quad (1)$$

In this notation (p,q), p indicates the order along the x-axis, and q the order along the y-axis. The TE and TM dispersions are then determined by the in-plane component of the wave vector, being along y or x, respectively. The angular dispersion of the bare array with PMMA is plotted in Figures S1(c) and S1(d). By varying the in-plane momentum along either k_y or k_x with the polarization of the incident beam along the x-direction, the transverse electric (TE) or transverse magnetic (TM) mode is excited. This is achieved in a Fourier microscope connected to a spectrometer with the slit of the spectrometer along the x or y axis of the particle array as illustrated in Fig. 2(a) of the manuscript. Fig S1 (e) shows the propagation length of the TE and TM SLRs, calculated from the width of the modes (1/Δk). The width is obtained by fitting a Lorentzian function to the dispersions in (c) and (d). The gap in the propagation length between ∼2.25 eV and ∼2.35 eV for the TE SLR corresponds to the gap in the dispersion at the point where the forward and backward propagating SLRs cross.

S2 Strong coupling of tetracene to TM SLR

Strong coupling between the TE-SLR and a 140 nm thick tetracene (Tc) crystal is shown in Fig.1(c) of the manuscript. However, the upper polariton band (UPB) was hardly visible, as it may be relatively broad and obscured by the vibronic progression. For TM polarization, however, the UPB is visible in the dispersion plot around 2.5 eV and $k_x \sim -5$ rad μm^{-1} , as shown in Fig. S2(a). The asymmetry in the dispersion of the LPB in this measurement is caused by the slight misalignment of the dipoles in the Tc with respect to the cavity field, resulting in a different dispersion for positive and negative in plane-momentum. A cut of this figure at $k_x = 5$ rad μm^{-1}, plotted in Fig. S2(b) with the black curve, shows the LPB at 2.24 eV and the UPB at 2.46 eV, evidencing the strong light-matter coupling. As a reference, the extinction of the bare Tc crystal is plotted with the gray dashed curve.

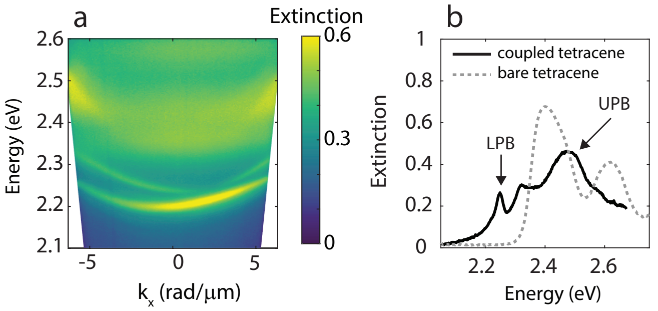

Figure S2: Angular dispersion of the TM mode of the particle array, strongly coupled to a 140 nm thick Tc crystal. The image is referenced by the extinction of the bare 140 nm thick Tc crystal. In (b) the extinction of the 140 nm Tc crystal is plotted with the gray dashed curve, while the extinction of the coupled system is plotted with the solid black curve.

S3 Dielectric function of tetracene

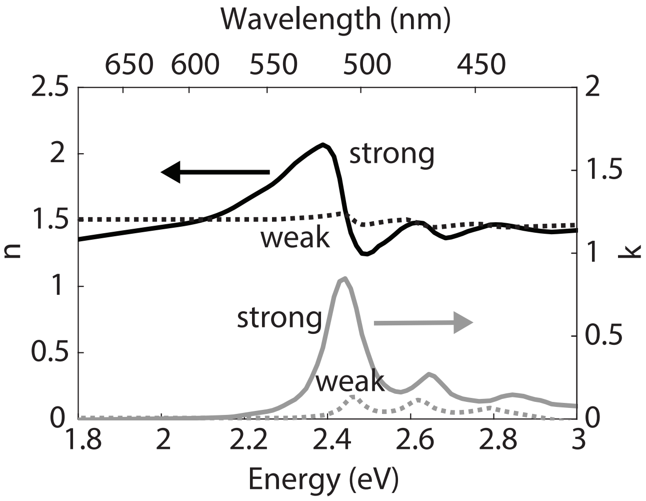

Figure S3: Solid gray and black curves correspond to the imaginary and real components of the refractive index of Tc along the strong axis used in the FDTD simulations throughout this manuscript. Dotted black and gray curves are the dielectric constants along the weak Tc axis. Values are adopted from Tavazzi et al.[2] and slightly shifted in energy to match the experimental absorption maximum.

S4 Propagation in weakly coupled tetracene

Figures 2(b) to (e) in the main manuscript show the Fourier images of exciton-polaritons propagation in strongly coupled Tc. A pronounced propagation length of the LPB and an energy shift of the emission intensity toward longer wavelengths for increasing distance from the excitation spot are observed. This is explained by the difference in group velocity and lifetime of the polariton at different energies. For the weakly coupled sample, the results are completely different.

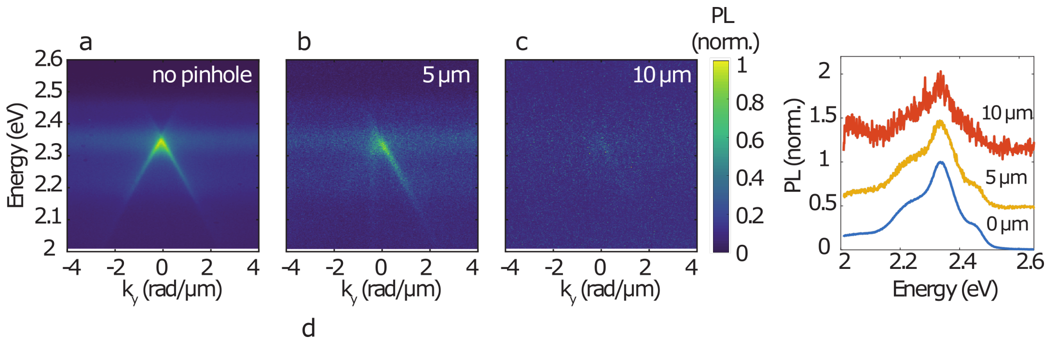

Figure S4: Emission collected directly *i.e.* without a pinhole for an orthogonal orientation of Tc dipoles with respect to the field (a). Emission collected through the pinhole at 5 (b) and 10 (c) µm away from the excitation spot. Integrated emission over the entire NA of the objective, showing no significant change in the spectrum for different distances (d).

When the crystal is rotated with respect to the array, Tc dipoles do no longer couple to the plasmonic array, resulting in a much weaker signature of the SLRs, which do no longer bend away from the exciton transition energy, as can be seen in Fig. S4(a). When we add the pinhole and move it 5 µm along the propagation direction in b) and c), we observe (as expected) that the SLR that is propagating in the opposite direction is no longer visible in the dispersion. The other SLR is still visible, albeit very weak. No difference in the shape of the SLR is visible at a distance of 5 µm from the excitation spot, which contrast to the strongly coupled case, where there is a

significant red shift of the LPB emission (see Fig. 2 of the manuscript). The emission is hardly visible at 10 μm from the excitation (Fig. S4(c)). The normalized angular integrated emission is plotted in Fig. S4(d) for the case without pinhole (blue curve), with a pinhole at 5 μm (orange curve) and with a pinhole at 10 μm (red curve) from the excitation spot. This figure stresses that the shape of the emission spectrum remains unchanged.

S5 Cross Section of the simulated and experimental propagation of the exciton polaritons

Figures 4(a) and (b) of the manuscript show the excellent qualitative agreement between the experimental propagation of polaritons probed by fluorescence after focused laser excitation and the simulated polariton propagation obtained from the propagation of the electric field squared $|E|^2$ of a dipole placed in the nanoparticle array. There is, however, a quantitative difference between the two results as the experimental propagation is longer than the simulated polariton propagation. This difference is shown more clearly in Fig. S5(a), where the blue dots show the experimental polariton propagation at an energy of 2 eV and the blue curve the simulated polariton propagation normalized at a distance of 1.5 wavelengths away from the dipole source. The orange dots show the experimental propagation at E=2.3 eV and the simulated propagation at that energy is plotted by the orange curve. The longer experimental propagation can be explained by

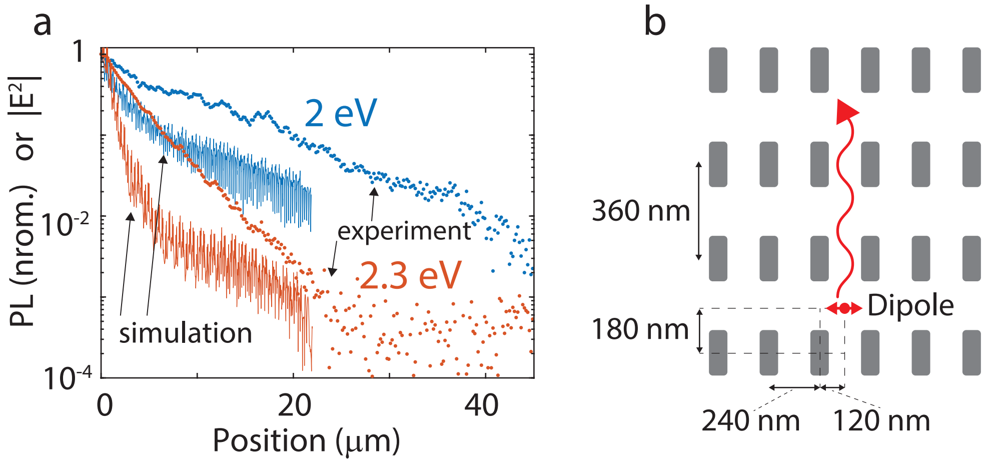

Figure S5: Experimental exciton-polariton propagation represented by blue and orange dots for energies of 2 and 2.3 eV, respectively (a). The simulated propagation is shorter and given by the blue and orange curves for 2 and 2.3 eV, respectively. All data sets are normalized. The position of the point dipole used in the FDTD simulations is shown in (b).

the relatively small size of the simulated particle array of 25x61 particles, which results in lower quality resonances. Another reason for the faster decay of the simulations could be that we use a point dipole is used as a source, while a finite sized laser spot is the source in the experiment. The position of the point dipole in the simulation is shown in (b) and it is at a height of 30 nm above the substrate.

S6 Simulated Electric Fields

The electric field in the plasmonic nanocavity is not homogeneous. To take this into account in the QM/MM simulations, the fields at the energy of the lower polariton were simulated using finite-difference in time-domain simulations (Lumerical) under normal incidence plane-wave illumation. The resulting fields are plotted in Fig. S6.

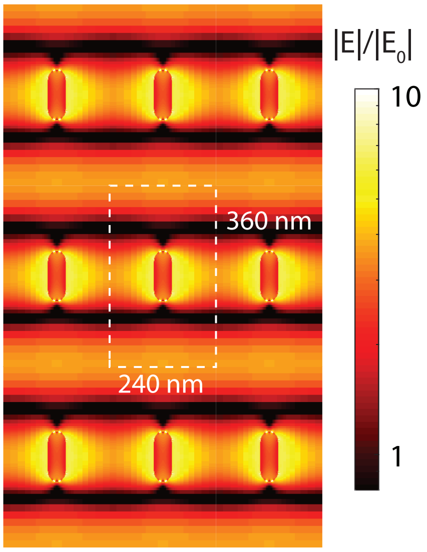

Figure S6: Logarithmic color map of electric fields at the energy of the SLR are shown for a silver nanoparticle of 42x100x40 nm^3 (WxLxH) in a lattice of 240x360 nm^2, 9 unit cells are plotted to show the extended nature of the SLR. The electric field amplitude is normalized by the incident amplitude.

S7 Fitting of the dispersions of the SLRs of the bare array

The dispersion of the SLRs arises from the coupling of Rayleigh Anomalies (RAs) and Localized Surface Plasmon Resonances (LSPRs) supported by the nanoparticle array. The measured SLRs dispersion can be thus described by the eigenvalues of the following Hamiltonian:

$$H = \begin{bmatrix} E_{LSPR} - i\frac{\gamma_{LSPR}}{2} & g_1 & g_2 \\ g_1 & E_{RA1} - i\frac{\gamma_{RA1(k_{||})}}{2} & 0 \\ g_2 & 0 & E_{RA2} - i\frac{\gamma_{RA2(k_{||}}}{2} \end{bmatrix} \quad (2)$$

where $\gamma_{LSPR} = 140$ meV is the losses of the LSPR extracted from the FWHM of the localized resonance; $g_1 = g_2 = 100$ meV are the coupling constants between the (0,-1) and (0,+1) TE RAs and the LSPRs, obtained by fitting this coupled oscillator model to the measured dispersion; $\gamma_{RA} = 40$ meV are the losses of the RAs, also a fitting parameter. The energy of the LSPRs (E_{LSPR}) is set at 2.62 eV in order to obtain the best fit to the dispersion. The energy of the RA depends on the incident wave vector and is calculated by:

$$E_{RA}(k_{||}) = \mp\frac{c}{n_{eff}}[k_y \mp \frac{2\pi}{a_y}], \quad (3)$$

with c the speed of light in vacuum, k_y the in-plane momentum along the y-direction, and $a_y = 360$ nm the period of the lattice in the y-direction. $n_{eff} = 1.48$ is the effective refractive index of the

surrounding medium, which is slightly lower than the index of the eagle glass substrate ($n \sim 1.51$) because of the small thickness of the PMMA layer on top of the array ($d \sim 200$ nm). The dispersions of the RAs are plotted with the black dotted diagonal lines in Fig. S7(a), while the horizontal black line indicates the energy of the LSPRs. The dispersions of the SLRs are plotted with the red dashed curves in Fig. S7(a). The color map in this figure corresponds to the measured angle dependent extinction spectrum. Figure S7(b) shows the dispersion of the three SLRs, while S7(c) gives the losses of each of these eigenmodes obtained from the imaginary component of the eigenvalues obtained by the diagonalization of the Hamiltonian. These results are used in the QM/MM simulations presented in the manuscript.

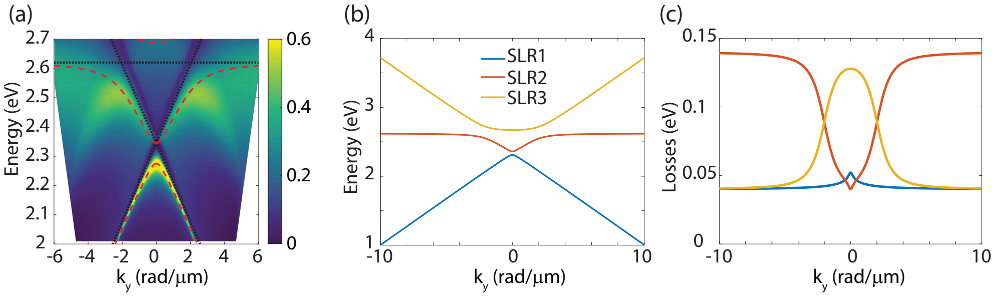

Figure S7: Fit to the dispersion of the SLRs (red dashed curves) on top of the experimental dispersion (color map) for the metallic nanoparticle array covered with PMMA (a). The black dotted curves correspond to the uncoupled dispersions of the LSPR and the RAs. Dispersion of the three SLRs, but for a larger range of angles (b). k_y-dependent losses of the three SLRs (c).

S8 Molecular Dynamics Simulations

S8.1 Simulation Model

Our atomistic Molecular Dynamics (MD) model is based on the Tavis-Cummings Hamiltonian of quantum optics, and models the interaction of N tetracene (Tc) unit cells strongly coupled to n_{mode} confined light modes:[3,4,5]

$$\begin{aligned}\hat{H}^{\text{TC}} \;=\; & \textstyle\sum_j^N h\nu_j(\mathbf{R}_j)\hat{\sigma}_j^+\hat{\sigma}_j^- + \sum_{k_y} \hbar\omega_{\text{cav}}(k_y)\hat{a}_{k_y}^\dagger\hat{a}_{k_y} + \\ & \textstyle\sum_j\sum_{k_y} \hbar g_j(k_y)\left(\hat{\sigma}_j^+\hat{a}_{k_y}\mathbf{f}_y(y_j) + \hat{\sigma}_j^-\hat{a}_{k_y}^\dagger\mathbf{f}_y^*(y_j)\right) + \\ & \textstyle\sum_i^N V_{\text{S}_0}(\mathbf{R}_i)\end{aligned} \quad (4)$$

Here, $\hat{\sigma}_j^+$ ($\hat{\sigma}_j^-$) is the operator that excites (de-excites) a Tc unit cell j from the electronic ground (excited) state $|\text{S}_0^j(\mathbf{R}_j)\rangle$ ($|\text{S}_1^j(\mathbf{R}_j)\rangle$) to the electronic excited (ground) state $|\text{S}_1^j(\mathbf{R}_j)\rangle$ ($|\text{S}_0^j(\mathbf{R}_j)\rangle$); $\mathbf{R}_j$ is the vector of the Cartesian coordinates of all atoms in unit cell j, centered at y_j; $\hat{a}_{k_y}$ ($\hat{a}_{k_y}^\dagger$) is the annihilation (creation) operator of an excitation of the SLR mode with wave-vector k_y; $\mathbf{f}_y(y_j) = e^{ik_y y_j}$ is the function describing the form of the quantized electromagnetic (EM) field modes, here taken to be that of plane waves with in-plane momentum k_y; $h\nu_j(\mathbf{R}_j)$ is the excitation

energy of unit cell j, defined as:

$$h\nu_j(\mathbf{R}_j) = V_{\mathrm{S}_1}^{\mathrm{mol}}(\mathbf{R}_j) - V_{\mathrm{S}_0}^{\mathrm{mol}}(\mathbf{R}_j) \tag{5}$$

with $V_{\mathrm{S}_0}^{\mathrm{mol}}(\mathbf{R}_j)$ and $V_{\mathrm{S}_1}^{\mathrm{mol}}(\mathbf{R}_j)$ the adiabatic potential energy surfaces of a Tc unit cell j in the electronic ground (S_0) and excited (S_1) state, respectively. The last term in Equation 4 is the total potential energy of the system in the absolute ground state (*i.e.*, with no excitations in neither the unit cells nor the SLR modes), defined as the sum of the ground-state potential energies of all Tc unit cells. We use a hybrid quantum mechanics / molecular mechanics (QM/MM) Hamiltonian to model the $V_{\mathrm{S}_0}^{\mathrm{mol}}(\mathbf{R}_j)$ and $V_{\mathrm{S}_0}^{\mathrm{mol}}(\mathbf{R}_j)$ potentials for the unit cells.[6]

As in Michetti and La Rocca,[5] we impose periodic boundary conditions in the y-direction, and thus restrict the wave vectors, k_y, to discrete values: $k_y = 2\pi n/L_y$ with $n \in \mathbb{Z}$ and L_y the length of the plasmonic array. With these approximations the molecular Tavis-Cummings Hamiltonian in Equation 4 can be represented as a $(N + n_{\mathrm{mode}})$ by $(N + n_{\mathrm{mode}})$ matrix with four blocks:

$$\mathbf{H}^{\mathrm{TC}} = \begin{pmatrix} \mathbf{H}^{\mathrm{mol}} & \mathbf{H}^{\mathrm{int}} \\ \mathbf{H}^{\mathrm{int}\dagger} & \mathbf{H}^{\mathrm{SLR}} \end{pmatrix} \tag{6}$$

The upper left block, $\mathbf{H}^{\mathrm{mol}}$, is a $N \times N$ matrix containing the single-photon excitations of the Tc unit cells. Because we neglect direct excitonic interactions between unit cells, the molecular block is diagonal, with elements labeled by the unit cell indices j:

$$H_{j,j}^{\mathrm{mol}} = \langle 0|\langle \mathrm{S}_0^1(\mathbf{R}_1)\mathrm{S}_0^2(\mathbf{R}_2)..\mathrm{S}_1^j(\mathbf{R}_j)..\mathrm{S}_0^N(\mathbf{R}_N)|\hat{H}^{\mathrm{TC}}|\mathrm{S}_0^1(\mathbf{R}_1)\mathrm{S}_0^2(\mathbf{R}_2)..\mathrm{S}_1^j(\mathbf{R}_j)..\mathrm{S}_0^N(\mathbf{R}_N)\rangle|0\rangle \tag{7}$$

for $1 \leq j \leq N$. Each matrix element of $\mathbf{H}^{\mathrm{mol}}$ thus represents the potential energy of Tc unit cell j (treated at the QM/MM level) in the electronic excited state $|\mathrm{S}_1^j(\mathbf{R}_j)\rangle$ while all other unit cells $i \neq j$ are in the electronic ground state $|\mathrm{S}_0^i(\mathbf{R}_i)\rangle$:

$$H_{j,j}^{\mathrm{mol}} = V_{\mathrm{S}_1}^{\mathrm{mol}}(\mathbf{R}_j) + \sum_{i \neq j}^{N} V_{\mathrm{S}_0}^{\mathrm{mol}}(\mathbf{R}_i) \tag{8}$$

The $|0\rangle$ in Equation 7 indicates that the single-photon Fock states of all SLR modes are empty.

The lower right block, $\mathbf{H}^{\mathrm{SLR}}$, is a $n_{\mathrm{mode}} \times n_{\mathrm{mode}}$ matrix (with $n_{\mathrm{mode}} = n_{\mathrm{max}} - n_{\mathrm{min}} + 1$) containing the single-photon excitations of the SLR modes, and is also diagonal:

$$H_{b,b}^{\mathrm{SLR}} = \langle 1_b|\langle \mathrm{S}_0^1(\mathbf{R}_1)\mathrm{S}_0^2(\mathbf{R}_2)..\mathrm{S}_0^N(\mathbf{R}_N)|\hat{H}^{\mathrm{TC}}|\mathrm{S}_0^1(\mathbf{R}_1)\mathrm{S}_0^2(\mathbf{R}_2)..\mathrm{S}_0^N(\mathbf{R}_N)\rangle|1_b\rangle \tag{9}$$

for $n_{\mathrm{min}} \leq b \leq n_{\mathrm{max}}$. Here $|1_b\rangle$ indicates the single-photon Fock state of SLR mode b with wave-vector $k_y = 2\pi b/L_y$. In these matrix elements, all Tc unit cells are in the electronic ground state (S_0), while SLR mode b is excited. The energy is therefore the sum of the SLR energy at k_y, and the molecular ground state energies:

$$H_{b,b}^{\mathrm{SLR}} = E_{\mathrm{SLR}}(2\pi b/L_y) + \sum_{j}^{N} V_{\mathrm{S}_0}^{\mathrm{mol}}(\mathbf{R}_j) \tag{10}$$

Here $E_{\mathrm{SLR}}(k_y)$ is an analytical expression for the dispersion of the $(0, +1)$ SLR, obtained as a non-linear fit to the dispersion determined experimentally (see section S7) and shown in Figure S7:

$$E_{\mathrm{SLR}}(k_y) = a_0 + \frac{1}{2}\left(a_1 + \sqrt{a_2^2 + a_3^2k_y^2} - \sqrt{a_1^2 + a_2^2 + 4a_4^2 + a_3^2k_y^2 - 2a_1\sqrt{a_2^2 + a_3^2k_y^2}}\right) \tag{11}$$

with different parameter sets a_i for SLRs 1 and 2 (see Table 1). SLR3, yellow curve in Figure S7,

is neglected since it lies significantly higher in energy than the S_1 state of Tc. An offset is added to parameter a_0 to compensate for a systematic deviation in the Tc excitation energy at the employed level of QM/MM theory (see below).

	a_0	a_1	a_2	a_3	a_4
SLR1	2.34927	-0.821206	-0.0411776	-0.163605	0
SLR2	-100.313	102.935	102.947	-6.97037	0.256437

Table 1: $E_{\mathrm{SLR}}(k_y)$ parameters

The two $N \times n_{\mathrm{mode}}$ off-diagonal blocks $\mathbf{H}^{\mathrm{int}}$ and $\mathbf{H}^{\mathrm{int}\dagger}$ in the multi-mode Tavis-Cummings Hamiltonian (Equation 6) describe interactions between the Tc unit cells and the SLR modes. These matrix elements are approximated as the overlap between the transition dipole moment of Tc unit cell j and the electric field of the cavity mode b at the center y_j of that unit cell:

$$H_{j,b}^{\mathrm{int}} = -\boldsymbol{\mu}_j(\mathbf{R}_j) \cdot \mathbf{E}(y_j)\langle 0_b|\langle \mathrm{S}_1^j|\hat{\sigma}_j^+ \hat{a}_b e^{i2\pi b y_j/L_y}|\mathrm{S}_0^j\rangle|1_b\rangle \tag{12}$$

for $1 \leq j \leq N$ and $n_{\min} \leq b \leq n_{\max}$. The position-dependent vacuum field strength $\mathbf{E}(y)$ in the nano-particle array is obtained by fitting the electric field distribution over the nano-particle along the long axis (Figure S6).

The multiple cavity-mode Tavis-Cummings Hamiltonian (Equation 6) is diagonalized at each time step of a MD simulation to obtain $N + n_{\mathrm{mode}}$ (adiabatic) polaritonic eigenstates ψ^m and energies E^m:

$$\psi^m = \sum_j^N \beta_j^m |\mathrm{S}_0^1\mathrm{S}_0^2..\mathrm{S}_1^j..\mathrm{S}_0^{N-1}\mathrm{S}_0^N\rangle|0\rangle + \sum_{b=n_{\min}}^{n_{\max}} \alpha_b^m |\mathrm{S}_0^1\mathrm{S}_0^2..\mathrm{S}_0^j..\mathrm{S}_0^{N-1}\mathrm{S}_0^N\rangle|1_b\rangle \tag{13}$$

All-atom trajectories of each Tc unit cell are computed by numerically integrating Newton's equations of motion. The total polaritonic wave function $\Psi(t)$ is coherently propagated along with the classical degrees of freedom as a time-dependent superposition of the $N + n_{\mathrm{mode}}$ time-independent *adiabatic* polaritonic states:

$$\Psi(t) = \sum_m^{N+n_{\mathrm{mode}}} c_m(t)\psi^m \tag{14}$$

where $c_m(t)$ are the time-dependent expansion coefficients of the time-independent polaritonic basis functions ψ^m defined in Equation 13. A Unitary Propagator in the *local* diabatic basis is used to evolve these coefficients along the trajectories of the N Tc unit cells.[7,8]

Spontaneous decay into the zero-photon subspace (*i.e.*, the ground state of the system) due to the emission of a photon is modelled as a first-order decay process of polaritonic states ψ^m that have a non-zero SLR contribution ($\sum_{b=n_{\min}}^{n_{\max}} |\alpha_b^m|^2 > 0$ in Equation 13).[9] The overall polaritonic decay rate κ^m is calculated as the sum over the products between the k_y-dependent SLR decay rates $\gamma_{\mathrm{SLR}}(k_y)$, (Figure S7c) and the weights of the total photonic contribution of mode b with the same $k_y = 2b\pi/L_y$ wave vector:

$$\kappa^m = \gamma(2\pi b/L_y)|\alpha_b^m|^2 \tag{15}$$

Thus, after an MD step Δt we multiply the population ($\rho_m = c_m^* c_m$) of state ψ^m by:

$$\rho_m(t + \Delta t) = \rho_m(t) \exp\left[-\sum_b^{n_{\text{mode}}} \gamma_{\text{cav}}(2\pi b/L_y)|\alpha_b^m(t)|^2 \Delta t\right] \tag{16}$$

Because $\rho_m = (\Re[c_m])^2 + (\Im[c_m])^2$, the change in the real and imaginary parts of the (complex) expansion coefficients $c_m(t)$ due to spontaneous photonic loss is:

$$\begin{aligned} \Re[c_m(t + \Delta t)] &= \Re[c_m(t)] \exp\left[-\tfrac{1}{2}\sum_b^{n_{\text{mode}}} \gamma_{\text{cav}}(2\pi b/L_y)|\alpha_b^m(t)|^2 \Delta t\right] \\ \Im[c_m(t + \Delta t)] &= \Im[c_m(t)] \exp\left[-\tfrac{1}{2}\sum_b^{n_{\text{mode}}} \gamma_{\text{cav}}(2\pi b/L_y)|\alpha_b^m(t)|^2 \Delta t\right] \end{aligned} \tag{17}$$

Simultaneously, the population of the zero-excitation subspace, or ground state ($\rho_0(t + \Delta t)$) increases as:

$$\rho_0(t + \Delta t) = \rho_0(t) + \sum_m \rho_m(t)\left(1 - \exp\left[-\sum_b^{n_{\text{mode}}} \gamma_{\text{cav}}(2\pi b/L_y)|\alpha_b^m(t)|^2 \Delta t\right]\right) \tag{18}$$

S8.2 Simulation Details

Starting from the tetracene (Tc) x-ray structure in the Cambridge Crystallographic Data Centre (CCDC), we constructed a unit cell containing 250 Tc monomers. Interactions between monomers within a unit cell were modeled with the 54A7 parameter set of the Gromos force field.[10] The topology was obtained from the ATB website.[11]

Prior to the QM/MM MD production runs, the simulation box, which contains 7500 atoms, was minimized with the steepest descent method. Subsequently, the entire system was equilibrated for 100 ns. The LINCS algorithm was used to constrain bond lengths and to enable a time step Δt of 2 fs. Temperature was maintained at 300 K with a stochastic thermostat ($\tau_T = 0.1$ ps).[12] A twin-range reaction-field was used to model the electrostatic interactions with cut-offs at 1.0 nm and 1.4 nm. Dispersion interactions were modeled with a Lennard-Jones potential truncated at 1.4 nm.

Snapshots from the equilibration trajectory were further equilibrated at the QM/MM level with a time step $\Delta t = 1$ fs. One Tc was described at the QM level of theory, while the remaining 249 monomers in the unitcell were modeled at the MM level. The QM subsystem was mechanically embedded and treated at the RHF/3-21G level of theory for the electronic ground state, while CIS/3-21G was used to model the singlet excited electronic state of a Tc monomer.

In total nine production runs were performed: three with 256 unit cells forming the crystal, three with 512 unit cells and three with 1024 unit cells. The unit cells were distributed equidistantly over the length of a one-dimensional periodic nano-particle array with a length of $L_y = 36$ µm to form a crystal. The crystals were strongly coupled to the (0,+1) SLR, which was modeled by 101 modes ranging from $n_{\min} = -50$, ($k_y = -8.73$ µm) to $n_{\max} = 50$ ($k_y = 8.73$ µm). All simulations were performed with Gromacs.4.5 version,[13] in which we have implemented the TC model described above in section S8.1 (code available for download from wwwuser.gwdg.de/∼ggroenh/gromacs-SLR.tar.gz).

S8.3 Simulation Analysis

Following Lidzey and co-workers, we computed the absorption spectrum of the Tc crystals strongly coupled to the $(0,+1)$ SLR of the nano particle array, as the total "visibility".[14] Visibility of a polaritonic state m is defined as the contribution of the $(0,+1)$ SLR modes to that state (*i.e.*, $I^m \propto \sum_b^{n_{\text{mode}}} |\alpha_b^m|^2$ with $n_{\text{mode}} = n_{\text{max}} - n_{\text{min}} + 1$). We thus computed the angle-resolved, or wave vector-dependent, one-photon absorption spectra of the strongly coupled Tc crystals as the convolution of the visibility and a Gaussian function:

$$I^{\text{abs}}(E,b) \propto \sum_i^s \left[\sum_m^{N+n_{\text{mode}}} \Delta E_i^m |\alpha_{b,i}^m|^2 \exp[-\frac{(E - \Delta E_i^m)^2}{2\sigma^2}] \right] \tag{19}$$

with $I^{\text{abs}}(E,b)$ the absorption intensity as a function of excitation energy E and in-plane momentum b ($k_y = 2\pi b/L_y$), s the number of trajectory frames included in the analysis (1001 frames), ΔE_i^m the excitation energy of polaritonic state ψ^m in frame i ($\Delta E_i^m = E_i^m - E_i^0$ with E_i^0 the energy of the ground state in frame i) and $\alpha_{b,i}^m$ the expansion coefficient of cavity mode b in polaritonic state m in frame i (Equation 13). A width of $\sigma = 0.05$ eV was chosen for all convolutions in this work. The spectra were plotted with Mathematica, version 11.3.[15]

The excitonic contribution to the polaritonic states were computed by projecting the N excitonic basis states (*i.e.*, $|\text{S}_0^1\text{S}_0^2..\text{S}_1^j..\text{S}_0^N\rangle|0\rangle$) onto the time-dependent wave function $\Psi(t)$ and summing over these basis states:

$$\rho_{\text{exc}}(t) = \sum_{j=1}^N \left| \langle \text{S}_0^1\text{S}_0^2..\text{S}_1^j..\text{S}_0^N|\langle 0|\Psi(t)\rangle \right|^2 = \sum_{j=1}^N \left| \sum_m^{N+n_{\text{mode}}} c_m(t)\beta_j^m \right|^2 \tag{20}$$

Likewise, the total SLR contributions was computed as the sum of the projections of the SLR modes basis states (*i.e.*, $|\text{S}_0^1\text{S}_0^2..\text{S}_0^N\rangle|1_b\rangle$)) onto the time-dependent wave function:

$$\rho_{\text{SLR}}(t) = \sum_{b=1}^{n_{\text{mode}}} \left| \langle \text{S}_0^1\text{S}_0^2..\text{S}_0^N|\langle 1_b|\Psi(t)\rangle \right|^2 = \sum_{b=1}^{n_{\text{mode}}} \left| \sum_m^{N+n_{\text{mode}}} c_m(t)\alpha_b^m \right|^2 \tag{21}$$

where $N + n_{\text{mode}}$ is the number of polaritonic states.

To monitor polariton propagation, we plotted the probability of the total wave-function as a function of distance y_j and time. $\Psi(y_j,t)$ is the sum of the amplitudes of its excitonic and SLR components: $\Psi(y_j,t) = \Psi^{\text{exc}}(y_j,t) + \Psi^{\text{SLR}}(y_j,t)$. The amplitude of the excitonic part at position y_j of unit cell j (with $y_j = (j-1)L_y/N$) arises from the contribution of the exciton j in that unit cell to all polaritonic eigenstates:

$$\Psi^{\text{exc}}(y_j,t) = \sqrt{\frac{N}{L_y}} \sum_m^{N+n_{\text{mode}}} c_m(t)\beta_j^m \tag{22}$$

Because SLR modes described by plane waves with wave-vector $k_{y,b}$ are delocalised in real space, the amplitude of the photonic part at position y_j of unit cell j results from interference of all SLR modes at that position. Thus, at position y_j, contributions of all SLR modes b in eigenstate m are first Fourier Transformed and subsequently summed over the polaritonic eigenstates:

$$\Psi^{\text{SLR}}(y_j,t) = \sum_m^{N+n_{\text{mode}}} c_m(t)\cdot\mathcal{FT}^{-1}\left[\Psi^{\text{SLR}}(k_y,t)\right] = \sum_m^{N+n_{\text{mode}}} c_m(t)\frac{1}{\sqrt{N}} \sum_b^{n_{\text{modes}}} \alpha_b^m e^{i2\pi(j-1)b/N} \tag{23}$$

Within the simulated time (100 fs), the implicit nanoparticle array length $L_y = 36$ μm is suf-

ficiently long to prevent self-interference of $\Psi(y,t)$ due to crossing of the periodic boundary at $y_{N+1} = y_1$.

To extract distance-dependent photoluminescence spectra in k_y-space from our QM/MM trajectories, we approximated pinholes by rectangular functions of width $\Delta y = 2$ μm centered at various positions y and accumulated the emission from the unit cells under that pinhole over the 100 fs simulation time. Emission at time t was obtained as follows: first, the excitonic parts (Equation 22) were convoluted with the rectangular function:

$$\Psi^{\text{exc}}_{\text{hole}}(y,t) = \sum_{y_j \geq y-\Delta y}^{y_j \leq y+\Delta y} \Psi^{\text{exc}}(y_j,t) \tag{24}$$

Likewise, the SLR part (Equation 23) was also convoluted:

$$\Psi^{\text{SLR}}_{\text{hole}}(y,t) = \sum_{y_j \geq y-\Delta y}^{y_j \leq y+\Delta y} \Psi^{\text{SLR}}(y_j,t) \tag{25}$$

$\Psi^{\text{pho}}_{\text{hole}}(y,t)$ was subsequently Fourier transformed from real space back into k_y space:

$$\Psi^{\text{SLR}}_{\text{hole}}(k_y,t) = \mathcal{FT}\left[\Psi^{\text{SLR}}_{\text{hole}}(y,t)\right] \tag{26}$$

such that the wave function can be written as $\Psi_{\text{hole}}(y,t) = \Psi^{\text{exc}}_{\text{hole}}(y,t) \otimes \Psi^{\text{SLR}}_{\text{hole}}(k_y,t)$. Because polaritonic eigenstates, ψ^m, form a complete set (Equation 13), the wave function under the pinhole at y and time t can be expressed in this basis:

$$\Psi_{\text{hole}}(y,t) = \sum_{m}^{N+n_{\text{modes}}} d_m \psi^m \tag{27}$$

The $N + n_{\text{modes}}$ expansion coefficients are obtained by projecting $\Psi_{\text{hole}}(y,t)$ on this basis:

$$d_m = \langle \psi^m | \Psi_{\text{hole}}(y,t) \rangle \tag{28}$$

Analogous to absorption (Equation 19), we approximated emission through the pinhole at time t as the "visibility" of the polaritonic states weighted by the expansion coefficients d_m :

$$I_{\text{hole}}(y,E,b,t) \propto \sum_{m}^{N+n_{\text{modes}}} |d_m|^2 |\alpha^m_b|^2 \exp\left[-\frac{(E-\Delta E^m)^2}{2\sigma^2}\right] \tag{29}$$

with $-50 \leq b \leq 50$, and $\sigma = 0.05$. The total distance-dependent photo-luminescence spectra were obtained by accumulating $I_{\text{hole}}(y,E,b,t)$ over the entire simulation:

$$I_{\text{hole}}(y,E,b) \propto \sum_{i}^{s} \sum_{m}^{N+n_{\text{modes}}} |d_{m,i}|^2 |\alpha^m_{b,i}|^2 \exp\left[-\frac{(E-\Delta E^m_i)^2}{2\sigma^2}\right] \tag{30}$$

with s the number of time steps (1001 in all simulations).

S8.4 Simulation results for different ensemble sizes

In Figure 3 in the main text, we showed results from simulations with 512 Tc unit cells. Results from simulations with 256 and 1024 Tc unit cells are shown in Figures S8 and S9. Comparing the propagation of the wave packets between the three crystal sizes, we observe that the distances covered by $\Psi(y,t)$ are independent of the number of unit cells included in the simulations. This result suggests that the propagation is determined by the group velocities in combination with the lifetimes of the lower polaritonic states. In contrast, because the lifetime of a strongly coupled

system depends on the size of the dark state manifold,[8] the rate of decay into the ground state is highest for the smallest system. However, as the propagation length depends on the lifetime of the bright polaritonic states, which are dominated by the SLR lifetime, rather than the overall lifetime of the whole system, this finite-size effect does not affect our conclusions about the transport mechanism.

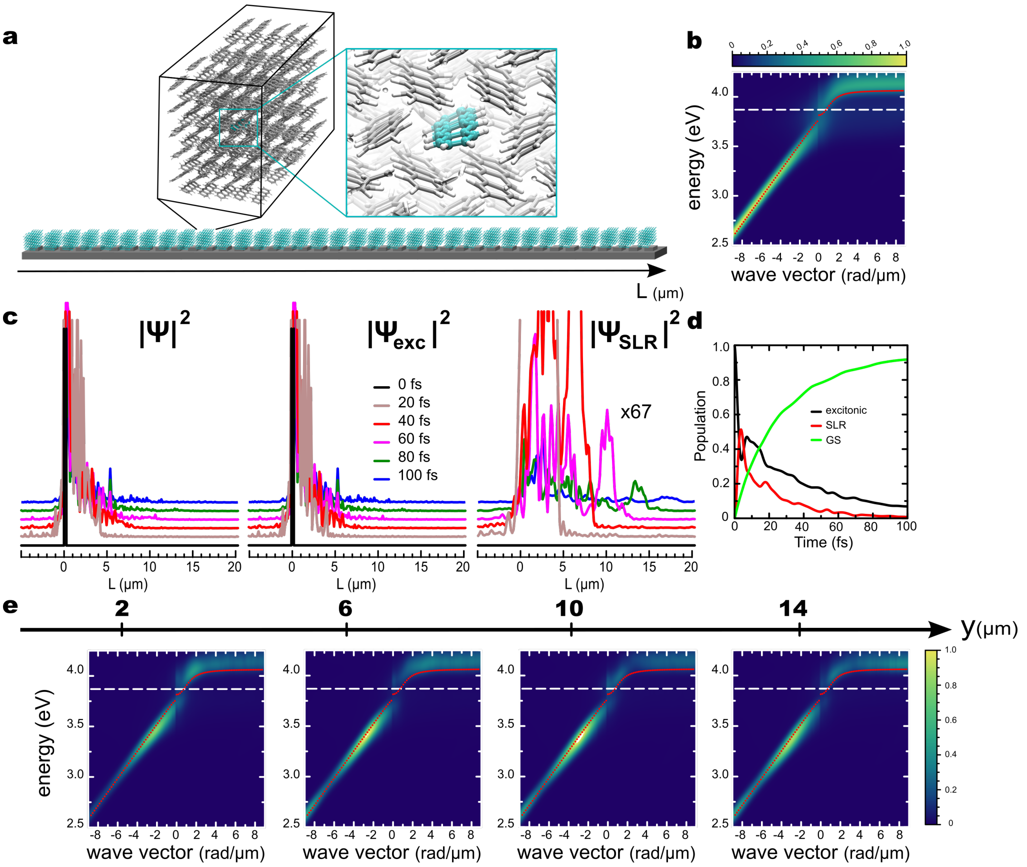

Figure S8: Schematic illustration of the simulation setup with the insets showing one of the 256 unit cells modelled in MD simulations (a). The Tc monomer described at the QM level is shown in ball-and-stick representation, while monomers in the MM subsystem are represented by sticks. Angle-resolved absorption spectrum when the crystal is strongly coupled to the modes of the $(0, +1)$ SLR (b). Wave-packet motion along the +y direction (c). For clarity, a vertical offset is added to wave packets at different time points. Time evolution of the excitonic (black) and photonic (red) fractions of polaritons in the Tc-SLRs strongly coupled system, as well as population of the ground state occupation (green), in which no photon is present (d). Angle-resolved photoluminesce spectra observed through pinholes located at various distances along the y-axis (e). Intensities in these plots are scaled by the same factor for each pinhole.

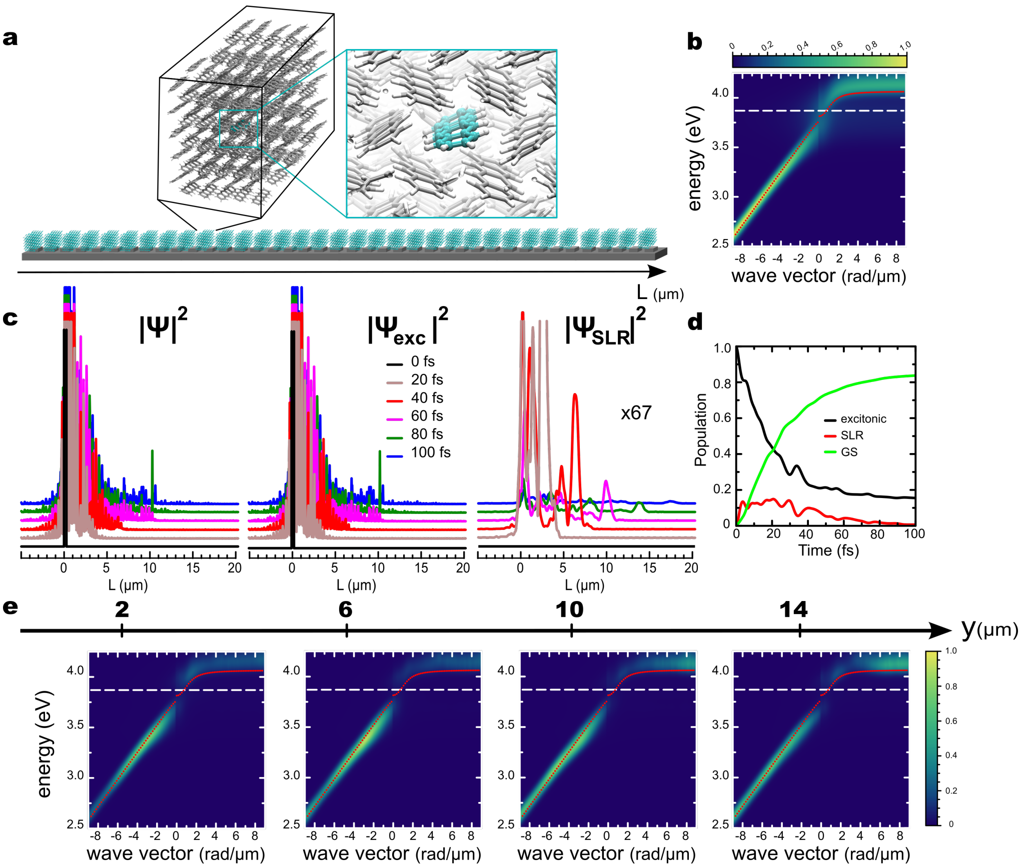

Figure S9: Schematic illustration of the simulation setup with the insets showing one of the 1024 unit cells modelled in MD simulations (a). The Tc monomer described at the QM level is shown in ball-and-stick representation, while monomers in the MM subsystem are represented by sticks. Angle-resolved absorption spectrum when the crystal is strongly coupled to the modes of the $(0, +1)$ SLR (b). Wave-packet motion along the +y direction (c). For clarity, a vertical offset is added to wave packets at different time points. Time evolution of the excitonic (black) and photonic (red) fractions of polaritons in the Tc-SLRs strongly coupled system, as well as population of the ground state occupation (green), in which no photon is present (d). Angle-resolved photoluminesce spectra observed through pinholes located at various distances along the y-axis (e). Intensities in these plots are scaled by the same factor for each pinhole.

S8.5 Interplay between polariton group velocity and lifetime

In spite of a much higher contribution from the short-lived SLR modes ($\sum_b^{n_{\text{mode}}} |\alpha_b(k_y)|^2$) in Figure S10a), polaritons at lower energy propagate over longer distances than polaritons at higher energies, where the polariton is dominated by contributions from the longer-lived excitons. The reason for such difference is that both polariton decay rate and group velocity depend on the energy (or k-vector, Figure S10b). Because the distance over which polaritons propagate depends on both lifetime and group velocity, and the group velocity is near zero for the longest living polariton states at $k_y > 4$ radμm^{-1}, the propagation is dominated by to the shorter-lived polaritons with the highest group velocities at lower k_y.

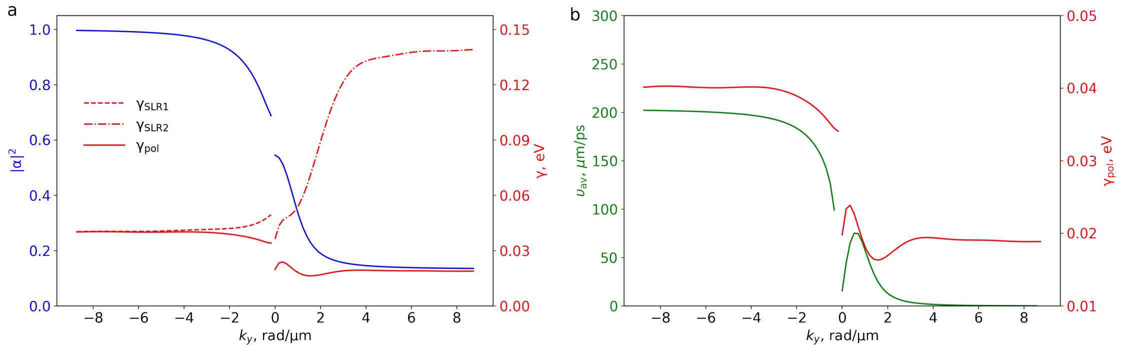

Figure S10: (a) Polariton decay rate $\gamma_{\rm pol}(k_y)$ (red solid line) obtained as a product of the SLR decay rate $\gamma_{\rm SLR}(k_y)$ (dashed and dash-dotted red lines for SLR1 and SLR2 modes, respectively) and the photonic fraction $|\alpha|^2$ (blue solid line) at each value of k_y. (b) Polariton decay rate (red line) and group velocity (green line) as functions of k_y.

References

[1] S. Murai, M. A. Verschuuren, G. Lozano, G. Pirruccio, S. R. K. Rodriguez, and J. Gómez Rivas. Hybrid plasmonic-photonic modes in diffractive arrays of nanoparticles coupled to light-emitting optical waveguides. *Optics Express*, 21(4):4250, 2013.

[2] S. Tavazzi, L. Raimondo, L. Silvestri, P. Spearman, A. Camposeo, M. Polo, and D. Pisignano. Dielectric Tensor of Tetracene Single Crystals: The Effect of Anisotropy on Polarized Absorption and Emission Spectra. *Journal of Chemical Physics*, 128(15):154709, 2008.

[3] E. T. Jaynes and F. W. Cummings. Comparison of quantum and semiclassical radiation theories with to the beam maser. *Proc. IEEE*, 51:89–109, 1963.

[4] M. Tavis and F. W. Cummings. Approximate solutions for an n-molecule radiation-field hamiltonian. *Phys. Rev.*, 188:692–695, 1969.

[5] P. Michetti and G. C. La Rocca. Polariton states in disordered organic microcavities. *Phys. Rev. B.*, 71:115320, 2005.

[6] A. Warshel and M. Levitt. Theoretical studies of enzymatic reactions: Dielectric, electrostatic and steric stabilization of carbonium ion in the reaction of lysozyme. *J. Mol. Biol.*, 103:227–249, 1976.

[7] G. Granucci, M. Persico, and A. Toniolo. Direct semiclassical simulation of photochemical processes with semiempirical wave functions. *J. Chem. Phys.*, 114:10608–10615, 2001.

[8] G. Groenhof, C. Climent, J. Feist, D. Morozov, and J. J. Toppari. Tracking polariton relaxation with multiscale molecular dynamics simulations. *J. Chem. Phys. Lett.*, 10:5476–5483, 2019.

[9] H.-L Luk, J. Feist, J. J. Toppari, and G. Groenhof. Multiscale molecular dynamics simulations of polaritonic chemistry. *J. Chem. Theory Comput.*, 13:4324–4335, 2017.

[10] Wei Huang, Zhixiong Lin, and Wilfred F. van Gunsteren. Validation of the gromos 54a7 force field with respect to β-peptide folding. *Journal of Chemical Theory and Computation*, 7(5):1237–1243, 2011. PMID: 26610119.

[11] Alpeshkumar K. Malde, Le Zuo, Matthew Breeze, Martin Stroet, David Poger, Pramod C. Nair, Chris Oostenbrink, and Alan E. Mark. An automated force field topology builder (atb) and repository: Version 1.0. *Journal of Chemical Theory and Computation*, 7(12):4026–4037, 2011. PMID: 26598349.

[12] G. Bussi, D. Donadio, and M. Parrinello. Canonical sampling through velocity rescaling. *J. Chem. Phys.*, 126:014101, 2007.

[13] B. Hess, C. Kutzner, D. van der Spoel, and E. Lindahl. Gromacs 4: Algorithms for highly efficient, load-balanced, and scalable molecular simulation. *J. Chem. Theory Comput.*, 4:435–447, 2008.

[14] D.G. Lidzey, D.C. Bradley, A. Armitage, S. Walker, and M.S. Skolnick. Photon-mediated hybridization of frenkel excitons in organic semiconductor microcavities. *Science*, 288:1620–1623, 2000.

[15] Wolfram Research, Inc. Mathematica, Version 11.3. Champaign, IL, 2018.
